# The nutrition and health risks faced by pregnant adolescents: Insights from a cross-sectional study in Bangladesh

**DOI:** 10.1371/journal.pone.0178878

**Published:** 2017-06-08

**Authors:** Phuong Hong Nguyen, Tina Sanghvi, Lan Mai Tran, Kaosar Afsana, Zeba Mahmud, Bachera Aktar, Raisul Haque, Purnima Menon

**Affiliations:** 1 Poverty, Health, and Nutrition Division, International Food Policy Research Institute, Washington, DC, United States of America; 2 FHI 360, Washington, DC, United States of America; 3 BRAC, Dhaka, Bangladesh; National Institute of Health, ITALY

## Abstract

Little is known about nutrition and well-being indicators of pregnant adolescents and the availability and use of nutrition interventions delivered through maternal, newborn, and child health (MNCH) programs. This study compared the differences between pregnant adolescents and adult pregnant women in services received, and in maternal and child nutrition and health conditions. A survey of 2,000 recently delivered women with infants <6 months of age was carried out in 20 sub-districts in Bangladesh where MNCH program is being implemented. Differences in service use and outcomes between pregnant adolescents and adult women were tested using multivariate regression models. The coverage of antenatal care and nutrition services was similar for adolescent and adult mothers. Compared to adult mothers, adolescent mothers had significantly fewer ownership of assets and lower decision making power. Adolescent mothers weighed significantly less than adult women (45.8 vs 47.1 kg, p = 0.001), and their body mass index was significantly lower (19.7 vs 21.3, p = 0.001). Adolescents recovered later and with greater difficulty after childbirth. Infants of adolescent mothers had significant lower height-for-age z-score (-0.89 vs -0.74, p = 0.04), lower weight-for age z-score (-1.21 vs -1.08, p = 0.02) and higher underweight prevalence (22.4% vs 17.9%, p = 0.04) compared to infants of adult women. In conclusion, this study confirms that adolescent pregnancy poses substantial risks for maternal and infant outcomes, and emphasizes that these risks are significant even where services during pregnancy are available and accessed. A focus on preventing adolescent pregnancy is imperative, while also strengthening health and nutrition services for all pregnant women, whether adult or adolescent.

## Introduction

Bangladesh contributes a substantial proportion of adolescent pregnancies to the global burden of premature motherhood. It is among one of the 10 countries with the highest prevalence of pregnancy among adolescent girls in both relative prevalence (40%) and absolute number (nearly 3 million) [1]. The health and social consequences of adolescent pregnancies are well documented [2,3]. The health consequences of adolescent pregnancy include the greater risk of anemia, low birth weight, preterm birth, maternal and neonatal mortality [4]. Adolescent girls also have high rates of complications from pregnancy, delivery, and unsafe abortion [5]. Social consequences include early school dropout, low empowerment, and risk of remaining poor [6]. Pregnancy during adolescence adds to the higher nutritional needs of adolescent girls as they are still growing and maturing. During the vulnerable period of adolescent growth and development, adequate nutrition plays a vital role in assuring a strong biological foundation for current and future health, well-being and productivity of women [5]. The recent Global Strategy for Women’s, Children’s and Adolescents’ Health focuses on critical population groups, such as adolescents and recommends greater investments in nutrition [7].

Based on currently available evidence, the report of the International Conference on Nutrition [8] recommends a set of interventions tailored for the nutritional needs of adolescents [2]. These include: promotion of diversified and adequate amounts of healthy food to meet micronutrient and energy needs; supplemental micronutrients (particularly iron, folic acid and calcium) where the requirements for these are not met through diet alone, provided through tablets or powders and if feasible, through food fortification; and prevention of adolescent pregnancy and promotion of adequate birth spacing. Additionally, World Health Organization (WHO) recommends promoting physical activity for energy balance to prevent obesity, addressing malaria and HIV AIDS where prevalent, and policies to protect food security for all populations [9].

Several program/policy issues related to adolescent pregnancy need to be addressed in each country context to respond to the global recommendations. A deeper understanding of how pregnant adolescents access health and nutrition programs and an analysis of the nutrition, well-being and health status of pregnant adolescents, is an important starting point for strengthening programming for adolescent pregnancies. In the context of an ongoing study to examine the feasibility of strengthening maternal nutrition interventions within an existing MNCH program (registered at ClinicalTrials.gov as NCT02745249), this paper examines differences in services received during pregnancy for these two groups and compares maternal and child nutrition and health conditions of pregnant adolescents with those of non-adolescents.

## Methods

### Study context

Data were drawn from the baseline household survey of a study which aims to test the feasibility of integrating a package of maternal nutrition interventions in a scaled up MNCH program implemented by a large non-governmental organization (BRAC) in Bangladesh. Two cadres of BRAC frontline workers deliver MNCH services: Shasthya Shebikas (SS) and Shasthya Kormis (SK). SS are community residents with 5–8 years of schooling, make visits to 150–200 households in their communities to identify new pregnancies, improve family health practices and sell a basket of health-related products including iron folic-acid (IFA) supplements, and attend deliveries. They are supervised by SK who have 10 or more years of schooling. Each SK supervises and supports 10 SS on average, serves a population of 1500–2000 households, provides messages on MNCH topics, conducts antenatal care (ANC) and postnatal care checkups, attends deliveries, offers essential newborn care, and refers complicated cases to government clinics. The standard nutrition interventions delivered through the MNCH program include nutrition education, selling of IFA supplements to pregnant women, deworming for women, and counseling on infant and young child feeding practices. Women are also able to receive IFA for free if they sought prenatal care at government clinics.

A cross-sectional survey was carried out between June and August 2015 in 20 sub-districts (*upazilas*) from four districts (Mymensingh, Rangpur, Kurigram, and Lalmonirhat) in which BRAC's existing rural MNCH project has been in place for more than 3 years. Within each sub-district, five unions and two villages within each union were randomly selected to yield a total of 200 villages (each had average size of 250 households). Within each village, a household census was conducted to create a list of mothers with infants <6 months of age. A total of 2,000 mother-infant pairs were selected for the survey using systematic sampling beginning with a random seed start point to yield the desired sample size per cluster.

Data were collected via face-to-face interviews using a structured questionnaire. Ethical approval was obtained from the Institutional Review Boards of the BRAC University in Bangladesh and the International Food Policy Research Institute, USA. Written informed consent was obtained from all women ≥18 years. For women <18 years of age, we obtained their assent and the permission of their guardians, i.e., their parents or husbands, to participate in the study.

### Measures

#### Coverage and use of maternal nutrition and health services

Mothers were asked about the antenatal services they received (ever received ANC, how early–the timing of the first visit, and total ANC visits), if they have been visited at home by BRAC frontline workers and the number of visits, content of counseling, specifically on nutrition topics such as diet and IFA/calcium supplements.

#### Maternal nutrition and health

Mother’s anthropometric measures were obtained using standard methods [10] by trained and standardized field staff. Weight was measured to the nearest 0.1 kg using electronic weighing scales. Height was measured to the nearest 0.1 cm using locally manufactured collapsible height boards. Body mass index (BMI) was calculated as weight (kg)/ height^2^ (meters).

In addition to standard indicators of illness following childbirth, we also collected information on maternal functional disability during the postpartum period. The measurement is based on the self-reported level of difficulty in performing self-care, child care, and household chores at four times after delivery (1–7 days, 8–15 days, 16–30 days, and 31–42 days) (S1 Table). These questions were developed based on WHODAS 2.0 manual for WHO Disability Assessment Schedule [11], then were pretested and adapted to be relevant for postpartum period and local context. We used factor analyses and assessed Cronbach’s alphas to determine if individual questions of maternal function disability belonged together. The reliability coefficients for different time points ranged from 0.93 to 0.97 (S2 Table), indicating high levels of agreement among questions. Additive scales were constructed in which one point was scored for each activity that the mother reported she could perform without difficulty at various times following delivery and the sum was used as the functional ability variable.

Maternal postpartum symptoms were measured based on mother’s recall of specific signs/ symptoms she experienced from birth to 6 weeks postpartum; a list of common signs/symptoms was used [12]. We also measured women’s decision making power (in food or medicine purchase, child care, mobility), ownership of assets (land, house, big and small animal).

#### Infant nutrition and health status

Infant weight was measured using electronic weighing scales (precise to 10g) and length was measured using locally manufactured length/height boards (precise to 1 mm), following a standardized method [10]. Weight and length were converted into height-for-age Z-scores (HAZ), weight-for-age Z-scores (WAZ), weight-for-height Z-scores (WHZ) [13]. Stunting, underweight and wasting were defined as <-2 Z-score of HAZ, WAZ, and WHZ, respectively [14].

Neonatal symptoms were measured based on mother’s recall of signs/symptoms her infant experienced from birth to 4 weeks (28 days) after birth. Her perception of her newborn’s birth weight as being very small or smaller than average was also assessed in the interview.

Breastfeeding practices were assessed to construct standard WHO indicators [15] for: 1) early initiation of breastfeeding (defined as the proportion of children born in the last 24 months who were put to the breast within one hour of birth); 2) pre-lacteal feeding (defined as the proportion of children born in the last 24 months who were given any food or liquid other than breast milk during the first three days after birth); and 3) exclusive breastfeeding (defined as the proportion of infants 0–5 months of age who were fed exclusively with only breast milk in the previous 24 hours); and 4) predominant breastfeeding (when the infant is given water, water-based drinks, fruit juice, ritual fluids, in addition to breast milk).

#### Independent variables

The main explanatory factor of interest was young maternal age, defined as mothers 13–19 years of age, or adolescent mothers. We created a variable for adolescent pregnancy (1, 0) using a cut-off of ≤19 years of age.

#### Covariates

We collected information on a wide range of variables that may relate to maternal and child health and nutrition. At maternal level, we controlled for maternal age, parity, education, occupation, use of antenatal care and nutrition services, contact with frontline health workers, and knowledge and practice of maternal nutrition recommendations. At the household level, we adjusted for household socioeconomic status (SES) and food security. Household SES was estimated using principal components analysis of data on housing conditions and asset holdings, and the first component derived from component scores was used to divide an SES score into tertiles [16,17]. Household food security was measured and calculated using FANTA/USAID’s Household Food Insecurity Access Scale [18] which provides information related to the household’s experience of food insecurity in the 30 days preceding the survey including anxiety and uncertainty about access, insufficient quality and quantity of intake. The households were categorized into two groups–food-secure and food-insecure (which included the mildly, moderately and severely food-insecure groups) [18].

### Statistical analysis

Differences in services received and in selected maternal and child nutrition and health conditions (referred to as “outcomes”) between pregnant adolescents and adult women were tested using multivariate linear regression models (for continuous variables) or logistic regression models (for categorical variables), controlling for covariates and taking into account the clustering of errors within and across *upazilas* (sub districts). All analysis was done using Stata 13. Statistical significance for differences between adolescent and adult mothers was considered at p < 0.05.

## Results

### Sample characteristics

The proportion of adolescents among recently delivered mothers was 20.2%. As expected, the average age of marriage and age at first birth was and parity were lower for adolescent mothers as compared with adults (Table 1). In both groups, marriage occurred at an average age of 16 to 17 years. The first child’s birth occurred at an average age of 18 to19 years. The majority of both adolescent and adult women were housewives. More adolescents had attended school and completed more years of schooling than adult women; however in both age groups more than 80% had dropped out before completing high school. Compared to adult mothers, significantly fewer adolescent mothers had decision making power on a variety of issues in the family and were less likely to have ownership of assets such as land, house or animals. The average total household size was ~5 members and adolescent women lived in households with more adults. No significant difference was found in household socioeconomic status. Food security ranged from 54 to 59% of households and was similar among adolescent and adult families.

**Table 1:** Maternal and household characteristics of recently delivered women, by age.

	Adolescent ≤19 years	Adult women >19 years
	(n = 404)	(n = 1,596)
	Mean ± SD/ Percent	Mean ± SD/ Percent
**Maternal characteristics**		
Age when first got married (y)	15.83 ± 1.32	16.80 ± 2.51**
Age having first child (y)	17.61 ± 1.19	19.33 ± 2.99**
Parity		
1 child	95.30	24.25
2 children	4.70	41.48
≥ 3 children	0.00	34.27
Education level		
Never attend school	3.22	13.72*
Primary school (grade1-5)	33.91	35.46
Middle school (grade 6–9)	46.29	35.78*
High school or higher	16.58	15.04
Occupation		
Household work/Housewife	95.54	88.41**
Others	4.56	11.69***
**Women’s decision making power**		
Buying animal source foods	26.24	44.17***
Buying cooking oil	31.93	51.32***
Buying medicine for yourself	45.05	59.09***
Buying medicine for the children	46.29	61.40***
What food is prepared every day	48.76	75.44***
Working to earn money	39.85	54.20***
Visiting other family members, friends or relatives	44.80	60.46***
Seeing a doctor or visiting a dispensary when pregnant	49.01	65.54***
Total decision making score	3.32 ± 2.82	4.72 ± 2.80***
**Women’s ownership of assets**		
Land	9.16	15.16**
House	18.07	23.43*
Animals like cows, horses, donkeys	20.54	30.58**
Small animals like hens, ducks, chickens, rabbits	36.39	56.70***
Total asset ownership score	0.81 ± 0.98	1.20 ± 1.05***
**Household characteristics**		
Household size	5.03 ± 2.10	5.12 ± 1.79
Number of adults (≥ 18 y)	3.01 ± 1.51	2.68 ± 1.32**
Household SES		
Low	32.49	33.59
Middle	25.44	35.31
High	42.07	31.10
Household food security	59.41	54.45

* p < 0.05

** p < 0.01

*** p < 0.001

### Use and awareness of health and nutrition services

The use of ANC services was similar for adolescent and adult mothers (Table 2). Almost all women ever received ANC services and nearly one half of received ANC from the first trimester of pregnancy. Two-thirds of women received prenatal visits at least 4 times during pregnancy and the average number of ANC visits was 6. IFA tablets were obtained free of cost by 40–50% of women and calcium was obtained free from government clinics by 30–33% of women. A majority of mothers (80–90%) were visited at home by BRAC health workers with an average of 2–3 visits during pregnancy. The levels of exposure to nutrition counseling and supplements during pregnancy were similar in adolescents and adult women. While nearly half of women reported being counselled on IFA and calcium consumption, less than 20% of them reported being counseled on weight gain and few had a weight gain chart.

**Table 2:** Mother’s access to health/ nutrition services and their knowledge and practice of maternal nutrition recommendations, by age.

	≤19 years	>19 years
	(n = 404)	(n = 1,596)
	Mean ± SD/ Percent	Mean ± SD/ Percent
**Received ANC services**		
Ever received ANC services	97.77	97.99
Received ANC from the first trimester	47.77	45.43
Received at least 4 ANC visits	66.58	67.23
Average number of ANC visits	6.22 ± 3.07	6.17 ± 2.95
IFA obtained free of cost	47.06	40.99
Calcium obtained free of cost	32.58	30.15
**Contact with SS/SK**		
Ever been visited at home by the SK	93.07	93.86
Number of times visited by the SK during this pregnancy	2.39 ± 1.88	2.60 ± 2.01
Ever been visited at home by the SS	80.45	86.78
Number of times visited by the SS during this pregnancy	3.46 ± 3.35	3.57 ± 3.10
**Counseling on nutrition topics**		
Eating additional amount of food	59.16	62.22
Taking IFA	47.77	50.94
Taking calcium	46.53	49.25
Taking weight	14.36	19.49*
**IFA supplement knowledge and practices**		
Ever heard about IFA tablets	99.50	99.50
Knowledge on benefit of IFA tablets (ranged 0–5)	1.75 ± 0.95	1.83 ± 1.01
Number of months pregnant women should take IFA tablets	5.97 ± 2.12	5.84 ± 2.05
Actual number of months RDW consumed IFA	3.25 ± 2.30	3.09 ± 2.24
**Calcium supplement knowledge and practices**		
Ever heard about calcium tablets	98.51	98.87
Knowledge on benefit of calcium tablets (ranged 0–3)	1.05 ± 0.62	1.18 ± 0.65***
Number of months pregnant women should take calcium tablets	5.81 ± 2.19	5.68 ± 2.11
Actual number of months RDW consumed calcium	2.75 ± 2.28	2.72 ±2.19
**Dietary diversity knowledge and practice**		
Knowledge on benefit of proper nutrition of pregnant women (ranged 0–4)	1.97 ± 0.81	2.10 ±0.85**
Number of food groups women should eat every day	5.25 ± 1.38	5.36 ± 1.40
% know women should consumed ≥5 food groups	70.54	72.37
**Delivery**		
Gave birth at home	57.92	62.91
Skilled birth attendance	81.44	82.64
C-section	21.78	22.37

* p < 0.05

** p < 0.01

*** p < 0.001

Knowledge of the benefits of consuming IFA was not significantly different among the age groups, but knowledge of the benefits of consuming calcium and proper diet was lower in adolescents compared to adult women. IFA and calcium were actually consumed for less than three months, as compared with the recommended six months during pregnancy, for both adolescents and adult women.

A large proportion of births took place in the home (58% among adolescents, 63% among adult women). Twenty-two percent of babies were delivered by C-section, with no difference between mother’s age groups.

### Health and nutrition outcomes

Although adolescent mothers were similar in height to adult mothers (Table 3), their body weight was significantly lower than adult women (45.8 vs 47.7 kg, p = 0.001), and their BMI was significantly lower (19.7 vs 21.3, p = 0.001). Adolescent mothers reported significantly lower scores for ‘postpartum functional abilities’ as measured by the ease of returning to household, self-care and child-care tasks at different periods of time postpartum (51.6 ±18.4 vs. 57.5 ±18.2), p<0.0001). No significant differences were found in maternal postpartum health symptoms.

**Table 3:** Maternal nutrition and health status, by mother’s age.

	≤19 years	>19 years
	(n = 404)	(n = 1,596)
	Adjusted mean ± SD/ percent	Adjusted mean ± SD/ percent
**Maternal nutritional status^1^**		
Height (cm)	149.88 ± 6.53	150.26 ± 5.70
Weight (kg)	45.78 ± 9.37	47.70 ± 8.17***
BMI	19.69 ± 3.08	21.25 ± 3.05***
BMI <18.5	29.87	18.80***
**Postnatal functional ability score^2^^,^^3^**		
Within 1–7 days	6.50 ± 4.43	7.76 ± 4.37***
Within 8–15 days	10.89 ± 5.86	12.69 ± 5.81***
Within 16–30 days	15.38 ± 5.90	17.19 ± 5.83***
Within 31–42 days	19.02 ± 5.47	20.11 ± 5.42***
Total functional ability score	51.60 ± 18.36	57.51 ± 18.17***
**Maternal postpartum symptoms^2^**		
Fever	32.15	32.46
Abdominal/uterine pain	42.46	43.45
Dysuria or flank pain	17.12	16.80
Headache	29.63	30.04
Fatigue/weakness/lethargy	48.12	51.47
Nausea/ Vomiting	4.18	6.48
Dizziness	22.78	22.64
Visual disturbance	8.65	9.11

^1^Model adjusted for education, occupation, parity, household SES and food security

^2^Model adjusted for education, occupation, parity, household SES and food security and C-section

^3^Scoring: The following household tasks were given scores of 2 = Could do without difficulty, 1 = could do with difficult, 0 = could not do at all. Not permitted to do is scored as missing. The tasks were: Take care of the newborn baby, Feed the baby, Bathe the baby, Wash the baby’s clothes, Prepare meals, Clean the house, Get water, Get to nearest health facility, Care for herself, Wash or bathe herself, Get dressed, Wash clothes and Use the toilet

*** p < 0.001.

Self-reported size of newborns tended to be lower among adolescents (Table 4). The age distribution of the infants was similar for the adolescent and adult mothers, but infants of adolescent mothers had significant lower height for age z-score (-0.89 vs -0.74, p = 0.04), lower weight for age z-score (-1.21 vs -1.08, p = 0.020) and higher underweight prevalence (22.4% vs 17.9%, p = 0.04) compared to infants of adult women. No significant differences were found in WHZ or wasting. Infant feeding practices and neonatal health symptoms were not significantly different between newborns of adolescent and older women.

**Table 4:** Infant nutrition, health status and infant feeding practices, by mother’s age.

	≤19 years	>19 years
	(n = 404)	(n = 1,596)
	Adjusted mean ± SD/percent	Adjusted mean ± SD/percent
**Perception of Birth weight^1^**		
Bigger than average	30.44	31.77
Average	44.31	46.37
Smaller than average	25.25	21.86*
**Child anthropometry^1^**		
HAZ	-0.89 ± 1.33	-0.74 ± 1.30*
WAZ	-1.21 ± 1.10	-1.08 ± 1.08*
WHZ	-0.68 ± 1.60	-0.75 ± 1.39
Stunting	15.89	15.07
Underweight	22.42	17.89*
Wasting	14.67	15.72
**Common neonatal symptoms^2^**		
Fever	34.28	36.97
Jaundice	13.04	12.47
Breathing difficulty	11.10	11.23
Skin infection	7.97	7.24
Vomiting/diarrhea	2.97	5.63
Umbilical cord infection	1.83	4.52
**Infant feeding practices^2^**		
Early initiation of breastfeeding	57.64	52.31
Prelacteal feeding	12.96	17.60
Exclusive breast feeding	70.80	66.22
Bottle feeding	8.92	11.02

^1^Model controlled for mother’s education, occupation, parity, BMI, ownership of asset, decision making power, and perception of low birth weight, household SES and food insecurity, child gender and child age month

^2^Model controlled for mother’s education, occupation, and parity, perception of low birth weight, household SES and food insecurity, child gender and child age month

* p < 0.05

## Discussion

Our findings show that adolescent pregnant mothers and older mothers have similar access to and utilization of ANC services in the context of MNCH services delivered by a large non-governmental organization in Bangladesh, indicating that BRAC’s MNCH program was effective in reaching both pregnant adolescents and older women. Despite receiving the same level of maternal health and nutrition services as adult pregnant women, adolescent mothers had lower maternal nutritional status as measured by BMI, took longer to recover after childbirth, and had children with poorer nutritional status. Our study suggests that adolescent pregnancy poses substantial risks for women’s and children’s outcomes, and emphasizes that these risks are significant even where services during pregnancy are available and accessed.

Most household characteristics (household size, socioeconomic status and food security) were similar between adolescent and adult families. The only key difference is that significantly fewer adolescents reported they had decision making power on a variety of issues in the family, including health and food. This could be a function of younger age and because they also had less ownership of assets. Lack of autonomy and economic independence affects the ability of women to access food resources and health care. Since husbands purchase foods for the household and adolescents do not have decision making power for foods, it could be useful to engage family members, including husbands of adolescents, to address some of these issues.

Significant differences between adolescents and women above 20 years were found in weight and BMI. Low BMI (below 18.5) was 52% higher among recently delivered adolescents as compared with women above 20 years. It is possible that pregnancy and lactation stalled the increases in body mass of adolescents, perhaps due to poor energy and nutrient supplies needed for body fat deposition and muscle mass, caused by the competition for nutrients between fetal growth needs and expanding tissues during pregnancy [19]. This curtailment has also been documented in adolescent growth in Bangladesh during pregnancy and lactation [20]. It is not possible to comment, however, on the basis of our study, whether additional nutritional support to pregnant adolescents can help to build muscle and fat stores and improve nutritional status.

Newborns of adolescent mothers had poorer nutritional status than newborns of their non-adolescent counterparts, including lower HAZ and WAZ, and higher underweight prevalence. This is in line with prior research in hospital setting in Bangladesh [21], where children of adolescent mothers were ~2 times more likely to be malnourished. It is likely that adolescent mothers did not have adequate reserves to enable adequate fetal growth due to competition with their own growth and development needs and smaller body size [4]. In another study, a longer follow up (until 48 months) also observed that children of adolescent mothers had lesser opportunities for immunization, were more prone to infectious diseases and had longer duration of hospitalization; all of these could also contribute to higher risk of undernutrition [21].

Compared with mothers over 20 years of age, adolescent mothers in our study reported significantly worse scores for ‘postpartum functional abilities’, particularly for activities related to taking care of her new born baby or doing some light household chores. This could be associated with the lower energy stores, reflected in higher proportion of low BMI in pregnant adolescents, or the extreme physiological changes that are induced by pregnancy. As maternal mortality reductions have been documented in a number of countries, the focus of maternal health policies and programs is shifting to address a wider range of maternal morbidities [22]. However, measuring morbidity related to poor nutrition is a challenge because nutritional indicators such as pregnancy weight gain, or biochemical indices of nutrient levels are not easily available in typical program settings, especially in adolescent populations. Our experience suggests that a functional disability score focused on assessing postpartum recovery in performing key tasks is feasible to adapt and implement in this context [22].

On a positive note, we found that exposure to the community health worker platform was high with no difference in the reach of these services between pregnant adolescents or adult women; more than 80 percent of women were visited at home by BRAC health workers and were counselled in various nutrition topics. The coverage of ANC services reported in our MNCH area study was higher compared to national average coverage (98% vs. 64% for receiving at least 1 ANC visit during pregnancy, and 67% vs. 31% for at least 4 ANC visits) [23]. The proportions of mothers with institutional delivery and having skilled attendant at birth were also higher in MNCH areas. The improvement in these coverages, together with other underlying factors, have contributed to the exceptional reduction in maternal mortality in Bangladesh [24]. The BRAC’s MNCH program [25] has been operating at a large scale in both rural (serving a population of 25 million in 14 districts,) and urban areas (serving a population of 7 million in 11 city corporations and 2 municipalities) since 2008 [26]. Previous research has shown that the behavior change components of the program were well accepted by the community, influencing them to seek MNCH services, improving community’s knowledge, attitudes, motivation and behavior change [27]; this likely facilitated the integration of nutrition interventions into this platform. The BRAC frontline health worker network is highly motivated and well-supervised, and cover a small catchment area, indicating that BRAC frontline health workers serve as good channels for conveying behavior-change messages and promoting good practices.

Despite high coverage of services and reasonably good awareness, nutrition practices such as weight gain monitoring and adherence with supplementation protocols and dietary diversity remained low, with no differences between adolescent and adult pregnant women. The measurement of weight during ANC contacts did not receive priority in this MNCH program. This is evidenced by a large proportion of mothers’ inability to recall messages given about adequate weight gain, and absence of a home-held record of weight taken during ANC visits. The total number of tablets consumed was about half of the nationally recommended 180 tablets for both IFA and calcium. Diet diversity also only practiced by half the pregnant women. The low number of IFA and calcium supplements consumed could be due to: 1) late registration so that supplies and counseling were started too late to complete the 6 months’ course of daily supplementation; 2) inadequate counseling on the daily use and management of potential side effects of IFA supplements; and 3) inadequate supplies because of limited willingness or ability to purchase supplements. This suggests a need to further emphasize behavior change interventions and other supporting strategies like free supplements to enable adolescents and adult women to follow recommended practices. A high number of reported ANC visits suggests that the content of the visits, rather than number of contacts, should be examined for further improvements in this MNCH program. Early initiation of breastfeeding was seen only in half of adolescent and adult mothers which left a substantial gap for further strengthening of nutrition interventions. Around 60% the deliveries took place at home, thus engaging support from family members and community birth attendants is critical to ensure timely initiation. To address the nutrition-related gaps identified in the current MNCH program, the Alive & Thrive project was explicitly designed to integrate intensified maternal nutrition interventions into the existing MNCH program platform (including intensified interpersonal counseling, community mobilization activities, distribution of free micronutrient supplements, and weight gain monitoring) since 2015. The impact evaluation results of this project will be published later when all data collection is completed.

To our knowledge, no other studies focus on the differences in maternal and child nutrition and health outcomes between adolescent and adult pregnancies in the context of large-scale MNCH programs. This study is particularly relevant as Bangladesh is one of just seven countries that account for half of all adolescent births worldwide [28]. The sample of adolescents and women is drawn from an area where an MNCH program has been active for over three years, thus providing an opportunity to understand how adolescents respond to MNCH services. A limitation of our study is the potential challenges to generalizability given the focused survey sample linked to an evaluation. However, the proportion of home deliveries (58–63%) and the percent of births delivered by C-section (22%) in this survey sample are similar to the recent nationally representative Demographic and Health Survey [29]. The proportion of underweight in infants below 6 months in the national survey was 19% which is compatible with 18% in older mothers in our study. Maternal functional disability was self-reported by RDW up to six months after delivery and therefore may suffer from recall bias. In addition, adolescent mothers are more likely to have had their first child with less child-care experience in the past, and therefore are more likely to report difficulties. However, we have included parity in the model to control for the number of births.

In conclusion, this study provides evidence that adolescent pregnancy poses substantial risks for women’s and children’s outcomes even when services during pregnancy are available and used. These differences are not due to broader differences in either household characteristics or health service access and use between adolescent and adult pregnant women, but are likely to be true differences stemming from premature motherhood. Policies and programs should continue, first and foremost, to focus on preventing adolescent pregnancy, while strengthening health and nutrition services for all pregnant women, whether adult or adolescent.

## Supporting information

S1 Table Postnatal functional disability questions. (DOCX)

S2 Table Factors and Cronbach's alpha of postnatal functional ability score. (DOCX)

S1 Data RDW_submit. (XLSX)

S1 Interview guide (PDF)

## Abbreviations

ANC: Antenatal care
BMI: Body Mass Index
HAZ: Height-for-age Z-scores
IFA: Iron folic acid
MNCH: Maternal, newborn, and child health
RDW: Recently delivered women
SES: Socioeconomic status
SK: Shasthya Kormis
SS: Shasthya Shebikas
WAZ: Weight-for-age Z-scores
WHO: World Health Organization
WHZ: Weight-for-height Z-scores
