# Supplementary material for: The nutrition and health risks faced by pregnant adolescents: Insights from a cross-sectional study in Bangladesh
Source: PLoS One. 2017 Jun 8;12(6):e0178878. doi: 10.1371/journal.pone.0178878 (PMC5464569; doi:10.1371/journal.pone.0178878)
Supplement: S1 Table — (DOCX) [file pone.0178878.s001.docx]

**S1 Table: Postnatal functional disability questions**

|  | Within 1-7 days of giving birth | Within 8-15 days of giving birth | Within 16-30 days of giving birth | Within 31-42 days of giving birth |
| --- | --- | --- | --- | --- |
|  | Could do without difficulty……………1  Could do with difficult…………… 2  Could not do at all….3 | Could do without difficulty……………1  Could do with difficult…………… 2  Could not do at all….3 | Could do without difficulty……………1  Could do with difficult…………… 2  Could not do at all….3 | Could do without difficulty……………1  Could do with difficult…………… 2  Could not do at all….3 |
| Were you able to take care of the newborn baby? |  |  |  |  |
| Were you able to feed the baby? |  |  |  |  |
| Were you able to bathe the baby? |  |  |  |  |
| Were you able to wash the baby’s clothes? |  |  |  |  |
| Were you able to prepare meals? |  |  |  |  |
| Were you able to clean the house? |  |  |  |  |
| Were you able to get water? |  |  |  |  |
| Were you able to get to nearest health facility? |  |  |  |  |
| Were you able to care for herself? |  |  |  |  |
| Were you able to wash or bathe herself? |  |  |  |  |
| Were you able to get dressed? |  |  |  |  |
| Were you able to wash clothes? |  |  |  |  |
| Were you able to use the toilet? |  |  |  |  |
