# Supplementary material for: The nutrition and health risks faced by pregnant adolescents: Insights from a cross-sectional study in Bangladesh
Source: PLoS One. 2017 Jun 8;12(6):e0178878. doi: 10.1371/journal.pone.0178878 (PMC5464569; doi:10.1371/journal.pone.0178878)
Supplement: S2 Table — (DOCX) [file pone.0178878.s002.docx]

S2 Table: Factors and Cronbach's alpha of postnatal functional ability score

|  | **Within 1-7 days** | **Within 8-15 days** | **Within 16-30 days** | **Within 31-42 days** |
| --- | --- | --- | --- | --- |
| Take care of the newborn baby | 0.73 | 0.83 | 0.82 | 0.85 |
| Feed the baby | 0.51 | 0.72 | 0.76 | 0.74 |
| Bathe the baby | 0.88 | 0.91 | 0.90 | 0.88 |
| Wash the baby’s clothes | 0.90 | 0.91 | 0.89 | 0.87 |
| Prepare meals | 0.45 | 0.69 | 0.76 | 0.76 |
| Clean the house | 0.92 | 0.90 | 0.89 | 0.86 |
| Get water | 0.89 | 0.87 | 0.89 | 0.83 |
| Get to nearest health facility | 0.68 | 0.90 | 0.89 | 0.81 |
| Care for herself | 0.71 | 0.89 | 0.89 | 0.88 |
| Wash or bathe herself | 0.84 | 0.89 | 0.88 | 0.89 |
| Get dressed | 0.67 | 0.79 | 0.81 | 0.80 |
| Wash clothes | 0.89 | 0.90 | 0.91 | 0.87 |
| Use the toilet | 0.62 | 0.72 | 0.73 | 0.68 |
| **Alpha** | 0.9595 | 0.9664 | 0.9521 | 0.9448 |
