# Supplementary material for: The nutrition and health risks faced by pregnant adolescents: Insights from a cross-sectional study in Bangladesh
Source: PLoS One. 2017 Jun 8;12(6):e0178878. doi: 10.1371/journal.pone.0178878 (PMC5464569; doi:10.1371/journal.pone.0178878)
Supplement: S1 Interview guide — (PDF) [file pone.0178878.s004.pdf]

Alive & Thrive Bangladesh Phase II- Maternal Nutrition
Baseline Survey 2015

Household Questionnaire

Recently Delivered Women

International Food Policy Research Institute (IFPRI)

Data Analysis and Technical Assistance

LIST OF MODULES

MODULE A. IDENTIFICATION 2

MODULE B. HOUSEHOLD COMPOSITION 4

MODULE C. OBSTETRIC HISTORY 5

MODULE D. USE OF ANTENATAL AND POSTNATAL SERVICES 5

MODULE E. CONSUMPTION OF SUPPLEMENTS 13

MODULE F. DIETARY INTAKE 14

MODULE G. IYCF PRACTICES 16

MODULE H. KNOWLEDGE ON MATERNAL NUTRITION 21

MODULE I. MEDIA HABIT 26

MODULE J. HOUSEHOLD SOCIO-ECONOMIC STATUS AND ASSETS 27

MODULE K. HOUSEHOLD FOOD SECURITY 30

MODULE L. WOMEN'S DIGNITY AND DECISION MAKING POWER 31

MODULE M. PHYSICAL AND MENTAL HEALTH OF THE RESPONDENT 33

MODULE N. POSTNATAL FUNCTIONAL DISABILITY AND POSTPARTUM SIGNS/ SYMPTOMS 34

MODULE O. DOMESTIC VIOLENCE 37

MODULE P. ANTHROPOMETRY 38

MODULE A. IDENTIFICATION

Name	Code
A.01 Household Number:	☐☐☐
A.02 Census number:	☐☐☐
A.03 Name of the recently delivery woman and member ID:..................	☐☐
A.04 Name of the Household Head:..	☐☐
A.05 Name of the father of the HH head [husband if female headed]:	☐☐
A.06 Para/Location/ Landmark :...	
A.07 Village:	
A.08 Mauza:	
A.9 Union/ Ward No:..	☐☐
A.10 Thana/Upazila:	☐☐
A.11 District:	
A.12 Mobile number:..	
A.13 Religion:	Muslim .... 1<br>Hindu .... 2<br>Christian.. 3<br>Buddhist.. 4<br>Other[specify] 5
A.14 Pregnant women delivered her baby ……….. days ago	☐☐ days<br>(*Interviewer: This can be maximum 179 days)*
A.15 Interviewer:...	☐☐
A.16 Supervisor:...	☐☐

	First Visit			Second Visit			
	Day	Month	Year	Day	Month	Year	
Date of Interview:	☐☐	☐☐	1 5	☐☐	☐☐	1 5	Signature of Supervisor

Starting time: **Ending time:**

DEFINITION OF HOUSEHOLD

A HOUSEHOLD IS A GROUP OF PEOPLE WHO LIVE TOGETHER AND TAKE FOOD FROM THE "SAME POT." IN OUR SURVEY, A HOUSEHOLD MEMBER IS SOMEONE WHO HAS LIVED IN THE HOUSEHOLD AT LEAST 6 MONTHS, AND AT LEAST HALF OF THE WEEK IN EACH WEEK IN THOSE MONTHS.

EVEN THOSE PERSONS WHO ARE NOT BLOOD RELATIONS (SUCH AS SERVANTS, LODGERS, OR AGRICULTURAL LABORERS) ARE MEMBERS OF THE HOUSEHOLD IF THEY HAVE STAYED IN THE HOUSEHOLD AT LEAST 3 MONTHS OF THE PAST 6 MONTHS AND TAKE FOOD FROM THE "SAME POT." IF SOMEONE STAYS IN THE SAME HOUSEHOLD BUT DOES NOT BEAR ANY COSTS FOR FOOD OR DOES NOT TAKE FOOD FROM THE SAME POT, THEY ARE NOT CONSIDERED HOUSEHOLD MEMBERS. FOR EXAMPLE, IF TWO BROTHERS STAY IN THE SAME HOUSE WITH THEIR FAMILIES BUT THEY DO NOT SHARE FOOD COSTS AND THEY COOK SEPARATELY, THEN THEY ARE CONSIDERED TWO SEPARATE HOUSEHOLDS.

GENERALLY, IF ONE PERSON STAYS MORE THAN 3 MONTHS OUT OF THE LAST 6 MONTHS OUTSIDE THE HOUSEHOLD, THEY ARE NOT CONSIDERED HOUSEHOLD MEMBERS. WE DO NOT INCLUDE THEM EVEN IF OTHER HOUSEHOLD MEMBERS CONSIDER THEM AS HOUSEHOLD MEMBERS.

EXCEPTIONS TO THESE RULES SHOULD BE MADE FOR:

CONSIDER AS HOUSEHOLD MEMBER

- A NEWBORN CHILD LESS THAN 3 MONTHS OLD.
- SOMEONE WHO HAS JOINED THE HOUSEHOLD THROUGH MARRIAGE LESS THAN 3 MONTHS AGO.
- SERVANTS, LODGERS, AND AGRICULTURAL LABORERS CURRENTLY IN THE HOUSEHOLD AND WILL BE STAYING IN THE HOUSEHOLD FOR A LONGER PERIOD BUT ARRIVED LESS THAN 3 MONTHS AGO.

DO NOT CONSIDER AS HOUSEHOLD MEMBER

- A PERSON WHO DIED VERY RECENTLY THOUGH STAYED MORE THAN 3 MONTHS IN LAST 6 MONTHS.
- SOMEONE WHO HAS LEFT THE HOUSEHOLD THROUGH MARRIAGE LESS THAN 3 MONTHS AGO.
- SERVANTS, LODGERS, AND AGRICULTURAL LABORERS WHO STAYED MORE THAN 3 MONTHS IN LAST 6 MONTHS BUT LEFT PERMANENTLY.

THIS DEFINITION OF THE HOUSEHOLD IS VERY IMPORTANT. THE CRITERIA COULD BE DIFFERENT FROM OTHER STUDIES YOU MAY BE FAMILIAR WITH, BUT YOU SHOULD KEEP IN MIND THAT YOU SHOULD NOT INCLUDE THOSE PEOPLE WHO DO NOT MEET THESE CRITERIA. PLEASE DISCUSS ANY QUESTIONS WITH YOUR SUPERVISOR.

MODULE B. HOUSEHOLD COMPOSITION

Member ID	Name<br>Now we would like to collect information on the different persons who usually live in your household.<br>Please tell me the name of all the persons who live in your house, starting with your name<br><br>(Start with pregnant/recently delivered woman)	Relationship to pregnant woman (code 1)	Gender<br>1= Male<br>2= Female	Age*		Marital status? (Code-2)	Occupation (Code-3)	Education (Highest class completed) (Code-4)	Monthly Income amount in taka**
				Years	Month				
B1	B2	B3	B4	B5.1	B5.2	B6	B7	B8	B9
1									
2									
3									
4									
5									

Code 1: Relationship to the respondent woman

Pregnant woman........................1
Spouse......................................2
Son or daughter 3
Father in-law 4
Mother in-law............................5
Grandchild 6
Father 7
Mother 8
Brother or sister........................9
Brother in law or sister in law ...10
Other relatives (including cousins)....................................11
Foster/step/adopted children 12
Not related................................13

Code-2 : Marital status

Unmarried.......... 1
Married............. 2
Widowed 3
Divorced 4
Separated 5

Code 3: Occupation

Farmer (Crops).................. 1
Agricultural day labor 2
Non Agricultural day labor 3
Service/Salaried worker..... 4
Small/cottage industry....... 5
Business/Traders 6
Rickshaw/van pulling........ 7
Other Self-employment 8
Household Work /Housewife 9
Maid servant 10
Student.............................. 11
Retired/Old age................. 12
Physically challenged........ 13
Jobless 14
Child (age <5)................... 15
Other 77

Code 4: Education

Never attended school 99
reads in class I.................................0
Completed class I...........................1
Put number of highest completed class. For example, if currently in class III, put 2 as class II is completed)
Completed Secondary School/Dakhil 10
Completed Higher Secondary/Alim 12
BA/BSC pass/Fazil 14
BA/BSC honors/Fazil (Hons) 15
MA/MSC/Kamil & above 16
SSC Candidate 22
HSC Candidate 33
Preschool class (general)................66
Hafezia/Kiratia/Nurani madrasa 67

For children under 5 years please record the complete age (years and months). Recording age in years only is sufficient for adults and children aged > 5 year

***** Write '0' for household members with no income***

MODULE C. OBSTETRIC HISTORY

C1.	What was your age when you first got married?	[__][__] years
C2.	How many times have you been pregnant?	[__][__] number of times
C3.	How many living children do you have?<br>*(all children including the current baby)*	[__][__] Children
C4.	What was your age when your first child was born?	[__][__] years
C5.	When was your previous birth before this baby?<br>*(if mothers do not remember exact dates, ask for immunization card or write month and year only)*	[__][__] / [__][__] / [__][__][__][__]<br>Day Month Year
C6.	Date of current delivery	[__][__] / [__][__] / [__][__][__][__]<br>Day Month Year

MODULE D. USE OF ANTENATAL AND POSTNATAL SERVICES

D.1. Postnatal Services

**Postnatal Services**		
D1.	Where did you give birth to this child?	Hospital/Private clinic/ health center .... 1>>D3<br>Maternity Centre .... 2<br>Own house .... 3<br>Father's house .... 4<br>Someone else's house .... 5<br>Others (specify) 77
D2.	Why did you NOT deliver at a health facility (hospital/private clinic)?	Costs too much .... 1<br>Facility not open .... 2<br>Too far/no transportation....................................... 3<br>Don't trust facility .... 4<br>Quality of service.. 5<br>No female provider at the facility .... 6<br>Not necessary .... 7<br>Not customary .... 8<br>Others (specify) 77
D3.	Who helped you during delivery of this child?<br><br>Probe to obtain all possible answers<br><br>(Multiple responses possible)	Doctor .... 1<br>Nurse/Midwife .... 2<br>FWA/HA.. 3<br>FWV .... 4<br>CHCP.. 5<br>BRAC SS .... 6<br>BRAC SK/CSBA.. 7<br>Other NGO workers.. 8<br>TTBA/Newborn health worker.............................. 9<br>TBA .... 10<br>Village Doctor .... 11<br>Homeopath doctor .... 12<br>Kabiraj/Herbal healer... 13<br>Spiritual healer .... 14<br>Mother/Mother-in-law .... 15<br>Other HH members.. 16<br>Neighbor/friends.. 17<br>Govt. or other CSBA 18

D4.	Was (NAME) delivered by caesarian, that is, they cut your belly open to take the baby out?	Yes .... 1<br>No .... 0<br>Do not know 99
D5.	After this child was born, how long did it take before any health care provider checked on the status of your child's health?	[__][__].[__] Hrs<br>[__].[__] Day<br>[__].[__] Week<br>Health was not checked........ 88>>D8
D6.	Who performed this check?	Doctor .... 1<br>Nurse/Midwife .... 2<br>FWA/HA........ 3<br>FWV .... 4<br>CHCP........ 5<br>BRAC SS .... 6<br>BRAC SK........ 7<br>Other NGO workers........ 8<br>TTBA/Newborn health worker........ 9<br>TBA .... 10<br>Village Doctor .... 11<br>Homeopath doctor .... 12<br>Kabiraj/Herbal healer........ 13<br>Spiritual healer .... 14<br>Mother/Mother-in-law .... 15<br>Other HH members........ 16<br>Neighbor/friends........ 17<br>Govt. or other CSBA 18
D7.	Where was this check performed?	Own house………1<br>BRAC Maternity Center………2<br>Medical College Hospital………3<br>District Hospital ………4<br>Upazila Health Complex………5<br>Pharmacy………6<br>Private clinic………7<br>Other NGO clinic………8<br>Community clinic………9<br>Family Welfare Center………10<br>At EPI center………11<br>Others………77
D8.	After this child was born, how long did it take before any health care provider, checked on the status of YOUR health?	[__][__].[__] Hrs<br>[__].[__] Day<br>[__].[__] Week<br>Health was not checked........ 88>>D11

D9.	Who performed this check?	Doctor ... 1<br>Nurse/Midwife ... 2<br>FWA/HA ... 3<br>FWV ... 4<br>CHCP ... 5<br>BRAC SS ... 6<br>BRAC SK ... 7<br>Other NGO workers ... 8<br>TTBA/Newborn health worker ... 9<br>TBA ... 10<br>Village Doctor ... 11<br>Homeopath doctor ... 12<br>Kabiraj/Herbal healer ... 13<br>Spiritual healer ... 14<br>Mother/Mother-in-law ... 15<br>Other HH members ... 16<br>Neighbor/friends ... 17<br>Govt. or other CSBA ... 18
D10.	Where was this check performed?	Own house ... 1<br>BRAC Maternity Center ... 2<br>Medical College Hospital ... 3<br>District Hospital ... 4<br>Upazila Health Complex ... 5<br>Pharmacy ... 6<br>Private clinic ... 7<br>Other NGO clinic ... 8<br>Community clinic ... 9<br>Family Welfare Center ... 10<br>At EPI center ... 11<br>Others ... 77
D11.	Did anyone help you with breastfeeding just after the birth of this child?	No ... 0>>D13<br>Yes ... 1<br>Do not know ... 99>D13
D12.	What did they do regarding breastfeeding?	Told to breasfeed the child ... 1<br>Showed the right way of positioning and attachment 2<br>Helped to place the baby on the breast ... 3<br>Others (specify) ... 77
D13.	What was the weight of the baby	[__].[__] kg<br>Don't know ... 99
D14.	When (NAME) was born, was s/he very large, larger than average, average, smaller than average or very small?	Very big ... 1<br>Bigger than average ... 2<br>Average ... 3<br>Smaller than average ... 4<br>Very small ... 5

D.2. ANTENATAL CARE

D15.	Have you received ANC during last pregnancy?	No .... 0>>D21<br>Yes .... 1<br>Do not know .... 99>>D21
D16.	From whom you received ANC during last pregnancy? (*multiple response possible)*<br>*(Interviewer: Probe to get all persons consulted)*	Doctor .... 1<br>Nurse/Midwife .... 2<br>FWA/HA .... 3<br>FWV .... 4<br>CHCP .... 5<br>BRAC SS .... 6<br>BRAC SK .... 7<br>Other NGO workers .... 8<br>TTBA/Newborn health worker .... 9<br>TBA .... 10<br>Village Doctor .... 11<br>Homeopath doctor .... 12<br>Kabiraj/Herbal healer .... 13<br>Spiritual healer .... 14<br>Mother/Mother-in-law .... 15<br>Other HH members .... 16<br>Neighbor/friends .... 17<br>Govt. or other CSBA 18
D17.	From where you received ANC during last pregnancy?	Own house .... 1<br>BRAC Maternity Center .... 2<br>Medical College Hospital .... 3<br>District Hospital .... 4<br>Upazila Health Complex .... 5<br>Pharmacy .... 6<br>Private clinic .... 7<br>Other NGO clinic .... 8<br>Community clinic .... 9<br>Family Welfare Center .... 10<br>At EPI center .... 11<br>Others 77
D18.	How many months pregnant were you when you first received ANC?	[__][__] number of months<br>Do not know 99
D19.	How many times did you did you receive ANC during last pregnancy?	[__][__] number of times<br>Do not know 99
D20.	During (any of your antenatal care visit(s), were you told about things to look out for signs that might suggest problems with the pregnancy?	No .... 0<br>Yes .... 1<br>Do not know 99
D21.	Have you ever been weighed during last pregnancy?	No .... 0>>D24<br>Yes 1

D22.	During your last pregnancy, who took your weight?	Doctor .... 1<br>Nurse/Midwife .... 2<br>FWA/HA .... 3<br>FWV .... 4<br>CHCP .... 5<br>BRAC SS .... 6<br>BRAC SK .... 7<br>Other NGO workers .... 8<br>TTBA/Newborn health worker .... 9<br>TBA .... 10<br>Village Doctor .... 11<br>Homeopath doctor .... 12<br>Kabiraj/Herbal healer .... 13<br>Spiritual healer .... 14<br>Mother/Mother-in-law .... 15<br>Other HH members .... 16<br>Neighbor/friends .... 17<br>Govt. or other CSBA 18
D23.	Where have you been weighed?	Own house .... 1<br>BRAC Maternity Center .... 2<br>Medical College Hospital .... 3<br>District Hospital .... 4<br>Upazila Health Complex .... 5<br>Pharmacy .... 6<br>Private clinic .... 7<br>Other NGO clinic .... 8<br>Community clinic .... 9<br>Family Welfare Center .... 10<br>At EPI center .... 11<br>Others 77
D24.	At what month of pregnancy were you first weighed during last pregnancy?	[__] number of months
D25.	Do you have a chart where your weight was recorded?	No .... 0<br>Yes 1
D26.	How many times was your weight measured during last pregnancy?	[__] number of times
D27.	How much weight did you gained during pregnancy? (Record from MN chart)	[__][__] kg
D28.	Did you receive any counseling or information about nutrition for pregnant/lactating women during last pregnancy?	No .... 0>>D28<br>Yes .... 1<br>Do not know .... 99>>D28

D29.	From whom did you receive this counselling or information?<br><br>*(multiple responses possible)*	Doctor .... 1<br>Nurse/Midwife .... 2<br>FWA/HA .... 3<br>FWV .... 4<br>CHCP .... 5<br>BRAC SS .... 6<br>BRAC SK .... 7<br>Other NGO workers .... 8<br>TTBA/Newborn health worker .... 9<br>TBA .... 10<br>Village Doctor .... 11<br>Homeopath doctor .... 12<br>Kabiraj/Herbal healer .... 13<br>Spiritual healer .... 14<br>Mother/Mother-in-law .... 15<br>Other HH members .... 16<br>Neighbor/friends .... 17<br>Govt. or other CSBA 18
D30.	Where did you receive this counselling?	Own house .... 1<br>District Hospital .... 2<br>Upazila Health Complex .... 3<br>Pharmacy .... 4<br>Private clinic .... 5<br>Community clinic .... 6<br>Family Welfare Center .... 7<br>At EPI Center .... 8<br>Others 77
D31.	During your last pregnancy, what topics were you counselled on about nutrition for pregnant women?<br>(*Interviewer: Do no prompt. Listen to what pregnant woman says and note 1 if what she says match with the options provided until the respondent says nothing else. Then check which ones in the list have not been marked yet and read those items to the respondents. If the respondent says yes, then note 1. Note 0 for all No responses)*	(see table below)
D32.	What messages did you receive on breastfeeding?	Initiate breastfeeding within the first hour of birth .... 1<br>Feed colostrum .... 2<br>Do not put anything in child's mouth (water, sugar water, honey, mustard oil, etc.) after birth .... 3<br>Feed only breastmilk to child for six months after birth. 4<br>Feed express breast milk if the mother goes out for long time .... 5<br>Others (specify) .... 77<br>Did not receive any counselling 88

D31 response grid:

	Unpromted	Prompted
Eat 5 variety of foods in addition to rice and dal		
Eat additional amount of food		
Taking weight		
Weight gain		
Nausea/vomiting		
Taking IFA		
Taking Calcium		
Taking Rest		
Avoiding Heavy Work		
Avoiding Tea/Coffee		
Others		
Don't know		

D33.	What messages did you receive on food variety?<br><br>*(multiple responses possible)*	Five types of food in addition to rice and thick dal......... 1<br>Consume Fish/Meat daily... 2<br>Consume Egg daily.. 3<br>Consume Milk/ Milk Product daily .... 4<br>Consume Dark Green leafy vegetable daily................... 5<br>Consume Yellow/ Orange fruit and vegetable daily .... 6<br>Consume thick daal everyday....................................... 7<br>Take nutritious snacks 2 times/day .... 8<br>Consume extra food (amount of a fist) with every meal . 9<br>Others (specify) .... 77<br>Did not receive any counselling 88
D34.	What messages did you receive on quantity of food?<br><br>*(multiple responses possible)*	A woman needs more energy and nutrients during pregnancy and lactation. .... 1<br>A variety of foods in additional amounts is required to meet the demands of the growing fetus...................... 2<br>Others (specify) .... 77<br>Did not receive any counselling 88
D35.	What messages did you receive on taking rest?<br><br>*(multiple responses possible)*	During pregnancy and postpartum period, a women should take rest at least for 2 hours after lunch .... 1<br>During pregnancy and postpartum period, a women should sleep for at least 8 hours at night .... 2<br>Taking rest is important for the growth of the baby........ 3<br>Taking rest improves weight gain of the mother .... 4<br>Others (specify) .... 77<br>Did not receive any counselling 88
D36.	What messages did you receive on gaining weight?<br><br>*(multiple responses possible)*	A women should gain 10-12 kg weight during pregnancy.1<br>Gaining weight indicates proper growth of the fetus ....2<br>Gaining weight indicates mother is taking adequate food .3<br>Others (specify) ....77<br>Did not receive any counselling 88
D37.	What messages did you receive on taking IFA tablet?<br><br>*(multiple responses possible)*	Take 1 tablet daily during pregnancy .... 1<br>Continue to take 1 tablet/day till 3 months postpartum... 2<br>IFA prevents anemia.. 3<br>IFA reduce risk of low birth weight baby .... 4<br>IFA reduce risk of maternal death due to hemorrhage ... 5<br>Do not take tea/ coffee after taking the iron tablet as it inhibits the absorption of iron....................................... 6<br>Others (specify) .... 77<br>Did not receive any counselling 88
D38.	What messages did you receive on taking Calcium tablet?<br><br>*(multiple responses possible)*	Take 1 tablet daily during pregnancy .... 1<br>Continue to take 1 tablet/day till 3 months postpartum... 2<br>Calcium helps in the development of bone and teeth of the baby .... 3<br>Calcium reduce risk of hypertension, eclampsia and pre-eclampsia .... 4<br>Others (specify) .... 77<br>Did not receive any counselling 88
D39.	Do you recognize this woman? (show photo of SK)	Yes... 1<br>No.. 0 >> D42

D40.	What kind of job does this woman do?<br><br>*[multiple responses possible]*	Check up on pregnant women .... 1<br>Checks up on children... 2<br>Gives health advice... 3<br>Gives nutrition child feeding advice .... 4<br>Gives advice on maternal nutrition .... 5<br>Conducts health forum...6<br>Helps during delivery.. 7<br>Give family planning advice .... 8<br>Provides IFA tablets .... 9<br>Provide calcium tablets .... 10<br>Gives health advice to husbands and family members.. 11<br>Fill-up MN chart... 12<br>Takes weight .... 13<br>Others (specify) .... 77<br>Don't know 99
D41.	In her capacity as a BRAC ____ , where have you seen this woman?	Visiting my home .... 1<br>In the para/village .... 2<br>Other (specify)... 77
D42.	Have you ever been visited at home by this woman?	Yes... 1<br>No 0
D43.	How many times did she visit you during this pregnancy?<br>(check MN chart and handbook)	[__] number of times
D44.	When was the last time she visited your home?	[___][___] days ago<br>[ ]months ago<br>Don't know/remember 99
D45.	Do you recognize this woman? (show photo of SS)	Yes... 1<br>No .... 0 >> go to next module
D46.	What kind of job does this woman do?<br><br>*[multiple responses possible]*	Check up on pregnant women .... 1<br>Checks up on children... 2<br>Gives health advice... 3<br>Gives nutrition child feeding advice .... 4<br>Gives advice on maternal nutrition .... 5<br>Conducts shasto forum.. 6<br>Helps during delivery.. 7<br>Others (specify) .... 77<br>Don't know 99
D47.	In her capacity as a BRAC ____ , where have you seen this woman?	Visiting my home .... 1<br>In the para/village .... 2<br>Other (specify)... 77
D48.	Have you ever been visited at home by this woman?	Yes... 1<br>No 0
D49.	How many times did she visit you during this pregnancy?	[__] number of times
D50.	When was the last time she visited your home?	[___][___] days ago<br>[ ]months ago<br>Don't know/remember 99

MODULE E. CONSUMPTION OF SUPPLEMENTS

		Iron Folic Acid	Calcium
E.1.	Did you ever consume ___________tablet during this pregnany?	Yes .... 1>>E3<br>No 0	Yes .... 1>>E3<br>No 0
E.2.	Why did you never consume the tablets	Never heard about them..……..1>> end IFA part<br>Don't know what they are for .. 2>>end IFA part<br>Don't have supply/never received3>>end IFA part<br>Possible side effects .... 4<br>Others.. 77<br>*Then go to Calcium questions*	Never heard about them .... 1<br>Don't know what they are for................... 2<br>Don't have supply/never received............. 3<br>Possible side effects .... 4<br>Others.. 77<br>*Then go to module F*
E.3.	Where did you get the tablets from?	Hospital/UHC .... 1<br>Doctor.. 2<br>Nurse/Midwife................................. 3<br>FWA/HA .... 4<br>FWV.. 5<br>CHCP .... 6<br>BRAC SS.. 7<br>BRAC SK .... 8<br>Other NGO workers .... 9<br>TTBA .... 10<br>TBA... 11<br>Village Doctor .... 12<br>Homeopath doctor........................... 13<br>Kabiraj/Herbal healer .... 14<br>Spiritual healer................................ 15<br>Pharmacy....................................... 16<br>Private clinic .... 21<br>Community clinic........................... 22<br>EPI .... 23<br>CSBA ....24<br>Others (specify) 77	Hospital/UHC .... 1<br>Doctor .... 2<br>Nurse/Midwife.. 3<br>FWA/HA .... 4<br>FWV .... 5<br>CHCP .... 6<br>BRAC SS .... 7<br>BRAC SK.. 8<br>Other NGO workers .... 9<br>TTBA .... 10<br>TBA .... 11<br>Village Doctor .... 12<br>Homeopath doctor................................. 13<br>Kabiraj/Herbal healer .... 14<br>Spiritual healer...................................... 15<br>Pharmacy.. 16<br>Private clinic... 21<br>Community clinic .... 22<br>EPI .... 23<br>CSBA ....24<br>Others (specify) 77
E.4.	Did you buy the tablets or did you get them for free?	Bought the tablets............................. 1<br>Received for free.............................. 2<br>Others(specify)............................... 77	Bought the tablets .... 1<br>Received for free.................................... 2<br>Others(specify) 77
E.5.	How many tablets did you buy/ get last month?<br>*Interviewer: Please ask the pregnant woman to show the tablet strip/bottle she got. Count the tablets and note it down.*	[__][__][__] number of tablets<br>Did not take any tablet last month<br>... 88>>E8	[__][__][__] number of tablets<br>Did not take any tablet last month<br>……………………………………-88>>E8
E.6.	Note down the composition of the tablet<br>*Interviewer: Look at the strip or bottle for composition of IFA and Calcium tablets and note down in the respective columns*	________ mg elemental iron OR<br>________ mg Ferrous Sulphate OR<br>________ mg Ferrous Fumerate<br><br>________ microgram Folic acid OR<br>________ mg Folic acid	_________mg Calcium
E.7.	Note down the tablets packaged<br>(surveyor: carry blister pack)	Bottled.. 1<br>blister pack...................................... 2<br>paper wrapped................................. 3<br>others (please specify) 77	Bottled.. 1<br>blister pack .... 2<br>paper wrapped.................................. 3<br>others (please specify)........................... 77

E.8.	How many tablets in total did you take during your current pregnancy?	[__][__][__] number of tablets	[__][__][__] number of tablets
E.9.	Do you consume IFA and Calcium tablet together at the same time or at different times?	Same time…………………………1<br>Different times……………………2<br>Not in any particular time………...3	Same time…………………………1<br>Different times……………………2<br>Not in any particular time………...3
E.10. .	When do you take the supplement (IFA or calcium) tablets?	Each night after dinner .... 1<br>Each morning with morning meal....... 2<br>Together with calcium tablet .... 3<br>Other (specify) 77	Each night after dinner .... 1<br>Each morning with morning meal....... 2<br>Together with calcium tablet .... 3<br>Other (specify) 77
E.11.	Have you noted down anywhere the number of tablets you take?	Yes .... 1<br>No 0	Yes .... 1<br>No 0
E.12.	Does any family member help you remember to take your tablets?	Yes .... 1<br>No .... 0>>E14	Yes .... 1<br>No .... 0>>E14
E.13.	Who in the family did help you remember to take your tablets?<br><br>(*multiple responses possible)*	Husband.. 1<br>Mother/Mother-in law...................... 2<br>Father/Father-in-law......................... 3<br>Brother-in-law.................................. 4<br>Sister-in-law.................................... 5	Husband .... 1<br>Mother/Mother-in law............................. 2<br>Father/Father-in-law................................ 3<br>Brother-in-law .... 4<br>Sister-in-law 5
E.14. .	Did you ever experience any side effects after taking the tablets?	Yes .... 1<br>No .... 0>> go to Calcium questions	Yes .... 1<br>No .... 0>>End module
E.15.	What kind of side effects did you experience due to taking the tablets?	Constipation.................................... 1<br>Swelling of abdominal area .... 2<br>Gas .... 3<br>Causes irritation/allergy.................... 4<br>Metallic taste.................................... 5<br>Do not know 99	Constipation... 1<br>Swelling of abdominal area .... 2<br>Gas .... 3<br>Causes irritation/allergy .... 4<br>Metallic taste .... 5<br>Do not know 99
E.16.	What did you do to manage the side effects?	Drink more waters............................ 1<br>Eating more fruits............................. 2<br>Eating more vegetables ....3<br>Changing the time to take tablets 4	Drink more waters............................ 1<br>Eating more fruits .... 2<br>Eating more vegetables ....3<br>Changing the time to take tablets....... 4
E.17.	Do you consider stop taking tables because of side effects?	Yes .... 1<br>No 0	Yes .... 1<br>No 0

MODULE F. DIETARY INTAKE

F.1. Dietary diversity of Lactating women

Interviewer: First ask if yesterday was a special day, like a celebration or feast day or a fast day where anyone in the HH ate special foods or where they ate more or less than usual or did not eat because they were fasting?

Was **yesterday** a special day where special kinds of foods were eaten? [ ] Yes..........1 No..........0

If yesterday was **not** a special day, then ask the respondent about the types of foods that they or anyone else in their household ate yesterday during the day and at night.

If yesterday **was** a special day, then ask the respondent to describe the foods (meals and snacks) consumed the **day before yesterday (or the last normal day)** during the day and night, whether at home or outside the home.

Sl. No	Questions	A. Lactating woman<br>Yes......1 No......0	B. Any HH Member<br>Yes......1 No......0
1.	CEREALS<br>Rice, bread made of wheat, puffed rice, pressed rice, noodles, or any other foods rice, wheat, maize/corn, *or other locally available grains*		

2.	VITAMIN A RICH VEGETABLES AND TUBERS<br>pumpkin, carrots, sweet potatoes that are orange and yellow inside		
3.	WHITE TUBERS AND ROOTS OR OTHER STARCHY FOODS<br>Potatoes, white yams, white sweet potato (not orange inside), potato crisps or other foods made from roots (not orange or yellow roots)		
4.	DARK GREEN LEAFY VEGETABLES<br>Dark green leafy vegetables, including spinach, red amaranth leaves, green amaranth, puishak, laushak, kumrashak, kolmishak, mustard leaves, yam leaves, koloishak (pea leaves), methishak (amaranth leaves), dhekishak, demishak etc		
5.	OTHER VEGETABLES<br>Other vegetables (e.g., squash, eggplant, green papaya, cauliflower, cabbage, onion, radish, sheem/borboti (beans),		
6.	VITAMIN A RICH FRUITS<br>Ripe mangoes, ripe papaya/pawpaw, jack fruit		
7.	OTHER FRUITS<br>Other fruits (e.g. banana, apples, guava, oranges, other citrus fruits, pine apple, shakalu, watermelon, olives, grapes, jambura (grapefruit) berries, kamranga, tamarind, plum		
8.	ANY BEEF, GOAT, LAMB, CHICKEN, DUCK, OR OTHER BIRDS, LIVER, KIDNEY, HEART, OR OTHER ORGAN MEATS		
9.	EGGS<br>Eggs of different birds – chicken, duck, etc.; with yolk, without yolk		
10.	FISH<br>Big/small fresh or dried fish or shellfish (e.g prawn, crab etc.)		
11.	ANY FOODS MADE FROM BEANS, PEAS, OR LENTILS<br>beans, peas, lentils, other pulses, soybeans, peas		
12.	MILK AND MILK PRODUCTS<br>Milk, cheese, yogurt or other milk products		
13.	OILS AND FATS<br>Oil, fats or butter added to food or used for cooking including ghee		
14.	SWEETS Sugar, molasses, honey, misti, cold drinks, chocolates, candies, biscuits		
15.	SPICES, CONDIMENTS, BEVERAGES<br>Spices (cumin, coriander, salt), condiments (pickles, chutney), coffee, tea, etc.		
16.	Tea/Coffee		

F.2 Assessments of food quantity using standard bowls

In this section interviewers will ask questions about the consumption of specific food items in different meal times. The quantity of food will be measured using 250ml bowls supplied to the FLWs by the program.
Interviewer: First ask if yesterday was a special day, like a celebration or feast day or a fast day where anyone in the HH ate special foods or where they ate more or less than usual or did not eat because they were fasting?
Was **yesterday** a special day where special kinds of foods were eaten? ☐ Yes..........1 No..........0

If yesterday was **not** a special day, then ask the respondent about the types of foods that they or anyone else in their household ate yesterday during the day and at night.
If yesterday **was** a special day, then ask the respondent to describe the foods (meals and snacks) consumed the **day before yesterday (or the last normal day)** during the day and night, whether at home or outside the home. While measuring quantity accept fraction (i.e. 1.5 bowl, 2.25 bow etc.).

**Have you taken this food item during:**	**Breakfast**		**Morning snack**		**Lunch**		**Afternoon snacks**		**Dinner**	
	Yes.....1<br>No......0	Quantity	Yes.....1<br>No......0	Quantity	Yes.....1<br>No......0	Quantity	Yes.....1<br>No......0	Quantity	Yes.....1<br>No......0	Quantity
Rice										

(bowl)										
Bread/Ruti (# of slices)										
Yellow/orange vegetables (Bowl)										
Dark green leafy veg (Bowl)										
Any other vegetable (Bowl)										
Egg (Number)										
Thick daal (Bowl)										
Yellow/orange fruits (Pieces)										
Citreous/sour fruits (Pieces)										
Any other fruits (Pieces)										
Milk (Cup)										
Milk product (Payesh, firni, curd, etc.) (Bowl)										
Big Fish (Pieces – match box size)										
Small fish (Bowl)										
Meat (Pieces – match box size)										
Biscuits/cakes (Pieces)										
Chanachur (Bowl)										
Puffed rice, *gur*, *khoi* (Bowl)										

MODULE G. IYCF PRACTICES

Sl #	Questions	Code
G.1.	How many hours/day after your last child's birth did you start breastfeeding?<br>(*Interviewer: Record 0 hour if the answer is immediately*)	[__][__].[__] Hrs<br>[__] Day
G.2.	Did you give the child colostrum?	Yes .... 1<br>No 0
G.3.	Was the child fed anything except breastmilk immediately after birth?	Yes .... 1<br>No .... 0>> G5<br>Don't know........ 99>> G5
G.4.	What was the child fed?<br><br>(multiple response possible)	Honey........ 1<br>Mustard oil........ 2<br>Plain water........ 3<br>Sugar/glucose water .... 4<br>Tea/coffee........ 5<br>Cow/goat milk .... 6<br>Other (specify)____________ .... 77<br>Do not remember 99

Sl #	Questions	Code
G.5.	Did you or anyone else give anything other than breastmilk to the child during the first 3 days after s/he was born?	Yes .... 1<br>No .... 0>>G7<br>Don't know .... 99>>G7
G.6.	During the first 3 days after the baby was born, what was given to the child by you or anyone else?<br>(Multiple response possible)	Honey .... 1<br>Mustard oil .... 2<br>Plain water .... 3<br>Sugar/glucose water .... 4<br>Tea/coffee .... 5<br>Cow/goat milk .... 6<br>Other (specify)____________ .... 77<br>Do not remember 99

WE WOULD LIKE TO ASK YOU ABOUT WHAT THE CHILD IS EATING NOW		
G.7.	Is the child still breastfeeding?	Yes ....1>>G10<br>No .... 0<br>Never .... 88>>G9
G.8.	At what age did you stop breastfeeding the child?	Month<br>Don't Know/cannot remember 99
G.9.	Why did you stop breastfeeding?<br><br>(Multiple response possible)	Problems with breast (pain) .... 1<br>Child not suck well .... 2<br>Not enough time to feed child .... 3<br>Child already grown up/ No need for breast feeding .... 4<br>Mother got pregnant .... 5<br>New baby born .... 6<br>Cracked nipples .... 7<br>Felt not enough breastmilk .... 8<br>Other (specify)________________. 77

G.10. At what age did you start giving the following liquids/foods to the child?

Note: if the mother fed her child any of the following within first 29 days (less than 1 month of age) then record "0" as the answer

1.	Water		Month<br>At "0" month of age .... 0<br>At "1" month of age .... 1<br>At "2" months of age .... 2<br>At "3" months of age .... 3<br>...<br>At "12" months of age .... 12<br>So on ....<br>Don't Know .... 99<br>Not given yet 88
2.	Other non breast milk liquids (sugar/glucose water, tea, fruit, juice etc.)		
3.	Cow/goat milk		
4.	Sooji/rice/gruel, etc.		
5.	Semi-solid foods (soft rice, khichuri, mashed potato, ripe banana, other mashed family foods, etc.)		
6.	Solid foods (such as rice, wheat, puffed/ pressed rice etc.)		
7.	Fish		
8.	Meat (chicken, mutton, beef, etc.)		
9.	Eggs		
10.	Legumes (pulse, peas, etc)		

	11. Vegetables	
	12. Snack foods (chanachur, chips, peanuts, biscuits)	
G.11.	Yesterday (during the day and the night), did you use a baby bottle to feed the child?	Yes.. 1<br>No.. 0
G.12.	How many times did you breastfeed the child yesterday, during the day and night?	[__][__] Number of times<br>Stopped breast feeding/Never breast fed.................... 88
G.13.	Other than breast milk, how many times did the child drink other milk, formula or yogurt yesterday, during the day and night?<br><br>DO NOT INCLUDE NUMBER OF TIMES THE CHILD WAS BREASTFED IN THIS QUESTION. THIS VARIABLE IS ONLY TO CAPTURE MILK OR MILK PRODUCTS **OTHER THAN BREAST MILK**.	[__][__] Number of times<br><br>Not given yet.. 88
G.14.	How many times did the child eat solid, semi-solid or soft foods other than liquids yesterday, during the day and night?<br><br>***Semi-solid*** *foods such as soft rice, mashed potato, ripe banana, other mashed family foods etc.* ***Solid*** *foods such as rice, wheat, puffed/pressed rice etc.*<br>*MEALS include both MEALS and SNACKS (other than trivial amounts)*	[__][__] Number of times<br><br>Not given yet.. 88

G.15. Yesterday (during the day and the night) did you give any of the following liquids to the index child?

Please describe everything that the child drank yesterday during the day or night, whether at home or outside the home.

a) Think about when the child first woke up yesterday. Did the child drink anything at that time? If yes: Please tell me everything the child drank at that time. Probe: Anything else? Until respondent says nothing else. If no, continue to Question b).

b) What did the child do after that? Did the child drink anything at that time?

If yes: Please tell me everything the child drank at that time. Probe: Anything else? Until respondent says nothing else.

Repeat question b) above until respondent says the child went to sleep until the next day.

Once the respondent finishes recalling foods eaten, read each food group where '1' was not circled, ask the following question and Circle '1' if respondent says yes, '0' if no and '99' if don't know.

After finishing first two columns, if you see none of the columns are marked '1' (yes) then move on to the third column. Answer to the third column must be recorded in months (e.g. if the mother says one year then record 12 months).

		**Has the child had this liquid yesterday?**<br>Yes…………1<br>No…… ……0<br>Don't know ..99	**Has the child ever had this liquid?**<br>Yes…………1<br>No…… ……0<br>Don't know ..99	**At what age of your child do you plan to start giving her this liquid?**<br>*Write age in months*<br>Don't know ..99
	1. Breast milk			
	2. Water			
	3. Baby formula (prepared food for child)			
	4. Any other kind of milk (powder, cow/goat milk etc.)			
	5. Fruit juice (made at home)			
	6. Fruit juice (purchased, packaged)			

	7. Water-based liquids, teas, sugar water, coffee			

G.16.	Please describe everything that the child ate yesterday during the day or night, whether at home or outside the home.<br>a) Think about when the child first woke up yesterday. Did the child eat anything at that time? If yes: Please tell me everything the child ate at that time. Probe: Anything else? Until respondent says nothing else. If no, continue to Question b).<br>b) What did the child do after that? Did the child eat anything at that time?<br>If yes: Please tell me everything the child ate at that time. Probe: Anything else? Until respondent says nothing else.<br>Repeat question b) above until respondent says the child went to sleep until the next day.<br>If respondent mentions mixed dishes like a PORRIDGE, sauce or stew, probe:<br>c) What ingredients were in that ***(MIXED DISH)***? Probe: Anything else? Until respondent says nothing else.<br>As the respondent recalls foods, underline the corresponding food and circle '1' in the column next to the food group. If the food is not listed in any of the food groups below, write the food in the box labeled 'other foods'. If foods are used in small amounts for seasoning or as a condiment, include them under the condiments food group.<br>Once the respondent finishes recalling foods eaten, read each food group where '1' was not circled, ask the following question and circle '1' if respondent says yes, '0' if no and '99' if don't know.<br><br>Yesterday during the day or night, did the child drink/eat any (FOOD GROUP ITEMS)?

	Foods	**Code**<br>**Yes=1**<br>**No=0**<br>**Don't know=99**
	1. Rice	
	2. Cereals such as wheat, pressed rice, puffed rice, suji	
	3. Purchased baby cereals (such as **Cerelac**, lactogen, Nan)	
	4. Legume: daal	
	5. Green leafy vegetables	
	6. Pumpkin, orange yam, orange-red-flesh sweet potatoe, carrots, tomato (vitamin-A rich)	
	7. Any other vegetables (starchy vegetables: potatoes, yam, plantain)	
	8. Ripe papaya or mango	
	9. Other fruits such as oranges, banana, grapefruits	
	10. Any other fruits	
	11. Beef, mutton	
	12. Chicken, duck, pigeon	
	13. Liver, heart, kidneys	
	14. Fish	
	15. Eggs	
	16. Peanuts, groundnuts, other nuts	
	17. Milk (non-human milk – cow, goat or powder)	
	18. Milk products (yogurt, rice pudding etc.)	
	19. Fat (oil, butter, ghee)	
	20. Chips or chanachur	
	21. biscuits	
	22. Bread or buns	
	23. Candies or chocolates	
	24. Pushtikona	
	25. Any iron containing tablet, syrup	
	26. Spices/condiments	
	27. Others (specify)	

**Difficulties in IYCF Practices**		
G.17.	Do you currently face any types of problems with feeding the child? Please think of any problems you might have faced in the last **one month**	Yes .... 1<br>No.. 0>>Next module

G.18.	What kind of problems do you currently face?<br><br>*(Multiple response possible)*	Problems with breast (pain) ....1<br>Child not suck well...2<br>Not able to breastfeed well ....3<br>Not enough time to feed child..4<br>Cracked nipples...5<br>Feel not enough breastmilk..6<br>Poor appetite ....7<br>Child does not want to eat...8<br>Child sick...9<br>Other (specify)___________ 77
G.19.	Did you seek help from anyone to help address this problem?	Yes .... 1<br>No.. 0>>Next module
G.20.	Who did you seek help from?<br><br>*(Multiple response possible)*	Doctor.. 1<br>Nurse/Midwife .... 2<br>FWA/HA .... 3<br>FWV... 4<br>CHCP .... 5<br>BRAC SS.. 6<br>BRAC SK .... 7<br>Other NGO workers .... 8<br>TTBA/Newborn health worker .... 9<br>TBA.. 10<br>Village Doctor... 11<br>Homeopath doctor .... 12<br>Kabiraj/Herbal healer .... 13<br>Spiritual healer.. 14<br>Mother/Mother-in-law... 15<br>Other HH members .... 16<br>Neighbor/friends .... 17<br>Govt. or other CSBA.................................... 18

MODULE H. KNOWLEDGE ON MATERNAL NUTRITION

Now I would ask you a few questions about your perceptions about diet and nutrition during pregnancy and postpartum

	**Questions**	**Code**
H.1.	Why is proper nutrition of pregnant women important?<br>*(Multiple response possible)*<br>(*Interviewer: Do no prompt. Listen to what pregnant woman says and note if what she says match with the options provided)*	For adequate weight gain of pregnant woman .... 1<br>Child inside the womb grows adequately/ healthy ....2<br>For a brainy child with bright future ....3<br>Quicker recovery after delivery..4<br>Extra costs due to doctors and medicine will be saved ....5<br>It is a good investment in future..6<br>To produce adequate breastmilk ....7<br>others (specify).. 77<br>Do not know 99
H.2.	How should a pregnant/lactating women eat in comparison with a non-pregnant woman to provide good nutrition to her baby and help him grow?<br>*(Multiple response possible)*	Eat 5 variety of foods in addition to rice and dal...................... 1<br>Eat fish/meat daily... 2<br>Eat egg daily .... 3<br>Take milk/ milk product daily.. 4<br>Eat green leafy vegetable daily .... 5<br>Eat yellow/orange vegetables/fruits daily .... 6<br>Take thick dal daily .... 7<br>Take nutritious food twice daily.. 8<br>Take one IFA tablet daily 9

		Take one Calcium tablet daily.. 10<br>Eat extra food (to the amount of a fist) with each meal .... 11<br>Other.. 77
H.3.	Have you heard about anemia?	Yes .... 1<br>No.. 0>>H5
H.4.	Can you tell me how you can recognize someone who has anemia?<br>*(Multiple response possible)*	Less energy/weakness .... 1<br>Paleness/pallor (pale color in eyes and palm) .... 2<br>More likely to become sick (less immunity to infections)..... 4<br>Other.. 5<br>Don't know 99
H.5.	Some beverages decrease iron absorption when taken with meals. Which ones?	Coffee .... 1<br>Tea... 2<br>Other .... 3<br>Don't know 99
H.6.	Have you ever heard about iron-folic acid (IFA) tablets?	Yes .... 1<br>No.. 0>>H10
H.7.	How many IFA tablets do you think a pregnant woman should take in one month?	[__] [__] Number of tablets<br>Don't know 99
H.8.	For how many months a pregnant woman should take IFA tablets?	[__] Months<br>Don't know 99
H.9.	Why do you think a pregnant woman should take iron folate tables?<br>*(Multiple response possible)*<br>(*Interviewer: Do no prompt. Listen to what pregnant woman says and note if what she says match with the options provided)*	To reduce the risk of anemia for pregnant women .... 1<br>To reduce risk of anemia for the child inside womb .... 2<br>To reduce the risk of low birth weight................................. 3<br>To help improve child's intelligence .... 4<br>To reduce the risk of excessive blood loss after delivery....... 5<br>To reduce risk of excessive blood loss during delivery .... 6<br>To make mother healthy/strong .... 7<br>Do not know 99
H.10.	Have you ever heard about calcium tablets?	Yes .... 1<br>No.. 0>>H14
H.11.	How many calcium tables do you think a pregnant woman should take in one month?	[__] [__] Number of tablets<br>Don't know 99
H.12.	For how many months a pregnant woman should take Calcium tablets?	[__] Months<br>Don't know 99
H.13.	Why do you think a pregnant woman should take calcium tables?<br>*(Multiple response possible)*	To recover the loss in pregnant woman's body..................... 1<br>To ensure adequate growth of child's bones and teeth .... 2<br>To reduce the risk of pre-eclampsia/eclampsia .... 3<br>Do not know 99
H.14.	How much rest should a pregnant woman take every day?	…….hours ……..minutes

Now I would ask some questions about foods to eat during pregnancy and postpartum.

H.15.	Do you know what *postpartum/ lactating woman* should eat every day?	Yes…...1<br>No……0	If yes, in what quantity each day?<br>*(using standard bowl/cup/ pieces)*
i	Rice		[__] [__] (# of bowl)
ii	Fish/Meat		[__] [__] pieces

iii	Egg		[__] [__] number
iv	Milk/ Milk products		[__] [__] bowl/glass
v	Dark green leafy vegetable		[__] [__] bowl
vi	Yellow/Orange vegetables/fruits (pumpkin, carrot, red amaranth, mango, jack fruit etc.)		[__] [__] bowl
vii	Thick daal		[__] [__] bowl
viii	Nutritious snacks		[__] [__] bowl/pieces

H.16 Now I would read out a few statements to you. You would kindly say if you ever heard this message or not. If you have heard this message then I would like to know form whom did you hear this message.

Questions		Have you heard this message?<br>Yes ....1<br>No...0>Next message	From whom/where did you hear? (code H)
1.	Proper diet every day during pregnancy ensures weight gain of pregnant woman		
2.	Proper diet every day during pregnancy ensures adequate growth of baby inside the womb		
3.	Proper diet everyday can ensure quick recovery of mothers after she gives birth to the child		
4.	Proper diet everyday during pregnancy can save costs on doctor and medicine for both mother and child		
5.	Nutritious food is not always expensive		
6.	Avoid hot foods (eg. ducks, pigeons, beef and Hilsha fish) during pregnancy		
7.	Daily consumption of fruits during pregnancy is essential to better health of a pregnant woman		
8.	Daily consumption of fish/meat/egg during pregnancy is essential because it ensures adequate growth and health of both child inside the womb and mother		
9.	Avoid some kinds of fish like Taki, Chanda, Puti and mrigal maach		
10.	During postpartum, take one IFA tablet everyday		
11.	During postpartum, take one Calcium tablet everyday		
12.	During postpartum, take at least two hours of rest every afternoon		
13.	Do not lay down on the bed, eat or cook during a lunar or solar eclipses		
14.	Pregnant women should consume at least one food item from 5 different food groups daily		
15.	Proper diet during pregnancy will ensure that the child will be brainy.		
16.	Avoid tea/coffee		
17.	Avoid alcohol/tobacco/betel leaf/betel nut		
18.	New born babies should be placed on mother's breast immediately after delivery		
19.	No water, honey or sugar water should be given to the new born babies after birth		
20.	Infants should be fed only breastmilk for the first six months		
21.	During pregnancy a woman should gain 10-12 kg weight		
22.	A PW should be weighted in each month		
23.	Lactating mother should eat only dry food during first 7 days after delivery		
24.	Pregnant women should not go outside house or visit graveyards after evening		

Code H			
Hospital/UHC……….….. 1	Other NGO workers………….9	Husband…..………..……….17	CSBS……………………24
Doctor…………………… 2	TTBA…………….…………..10	Mother/Mother-in-law…………………………..18	Radio…………………….25
Nurse/Midwife……….…. 3	TBA…………………………..11	Other HH members………....19	Books/Newspaper/ Poster/Billboard……..….26
FWA/HA………….…….. 4	Village Doctor………………..12	Neighbor/friends……..…….20	Internet/website…......….27
FWV…………………….. 5	Homeopath doctor………..…..13	Private clinic……………..…21	Jatra/Pala/Cinema…........28
CHCP…………………… 6	Kabiraj/Herbal healer………...14	Community clinic………..…22	Video show...........29
BRAC SS……………….. 7	Spiritual healer……………..…15	EPI…………….…………….23	Others (specify)………....77
BRAC SK…………….... 8	Pharmacy…………….………16		

H.17 Perceptions and drivers of behavioral change

Please tell us if you agree with the following statements. The level of agreement runs from 1-5 with 5 is strongly agree and 1 is strongly disagree

	**Statements**	**Yes, agree**…………….1<br>**No, disagrer**…………2
1.	My consuming right types and amount of food during pregnancy is extremely important for my health and my unborn child	
2.	My consuming right types and amount of food during pregnancy is extremely important for my unborn child's brain/education and ability to earn	
3.	I can manage to follow the recommendations of 5 varieties of food to be consumed during pregnancy	
4.	I can manage to follow the recommendations of adequate amounts of food to be consumed during pregnancy	
5.	My family members and community people will be angry if I consume the right types and amounts of food during pregnancy	
6.	I cannot consume the recommended types and amounts of food as we are poor people	
7.	It is too costly to obtain the recommended types and amounts of foods for my consumption during pregnancy	
8.	It is a good use of our family's money to ensure the right types and amounts of foods during pregnancy and it contributes to the future welfare of the child and family	
9.	In my family and community I am expected to consume so many varieties and such large amount during pregnancy	
10.	My husband knows the importance of proper nutrition for mother during pregnancy	
11.	My husband does not purchases diversified nutritious foods and does not ensure that I have these foods available	
12.	My husband reminds and encourages me to consume the recommended quantity of diversified foods daily	
13.	My husband helps me to ensure that there are enough tablets of IFA and Calcium at home	
14.	My husband reminds me to take one tablet of IFA and on tablet of Calcium daily	
15.	My husband does not remind /helps me to take rest for 2 hours during the day in addition to sleeping at night	
16.	My husband and family members make me lifting heavy work load during pregnancy	
17.	My husband reviews my weight gain chart and helps me find ways to gain enough weight during pregnancy	
18.	My husband calls the health worker on mobile if I have any difficulties to do any of the above	

MODULE I. MEDIA HABIT

I.1.	Do you ever watch TV?	Yes .... 1<br>No .... 0>>I.5
I.2.	How often do you watch TV?	Daily (7 days a week) .... 1<br>2 to 6 days a week .... 2<br>Once a week .... 3<br>Once every two weeks .... 4<br>Once a month .... 5<br>Rarely 6
I.3.	What time of the day do you watch TV?<br>*(Multiple response possible)*	0:00 – 5.59 hrs .... 1<br>6:00 – 1.59 hrs .... 2<br>12:00 – 17:59 hrs .... 3<br>18:00 – 23.59 hrs 4
I.4.	Which programmes do you watch commonly?<br><br>*(Multiple response possible)*	News .... 1<br>Music .... 2<br>Children's program .... 3<br>Religious program .... 4<br>Sports .... 5<br>Soap opera .... 6<br>Health/ disease programs .... 7<br>Movie .... 8<br>Other (specify) 77
I.5.	Do you ever listen to the Radio?	Yes .... 1<br>No .... 0>> end module
I.6.	How often do you listen to the Radio?	Daily (7 days a week) .... 1<br>2 to 6 days a week .... 2<br>Once a week .... 3<br>Once every two weeks .... 4<br>Once a month .... 5<br>Rarely 6
I.7.	What time of the day do you listen to the Radio?<br>*(Multiple response possible)*	0:00 – 5.59 hrs .... 1<br>6:00 – 1.59 hrs .... 2<br>12:00 – 17:59 hrs .... 3<br>18:00 – 23.59 hrs 4
I.8.	Which programmes do you listen to commonly?<br><br>*(Multiple response possible)*	News .... 1<br>Music .... 2<br>Children's program .... 3<br>Religious program .... 4<br>Sports .... 5<br>Soap opera .... 6<br>Health/ disease programs .... 7<br>Other (specify) 77

MODULE J. HOUSEHOLD SOCIO-ECONOMIC STATUS AND ASSETS

Household construction

J.1.	Do you own the house you live in?	Owns house .... 1<br>Rents .... 2<br>Free housing .... 3<br>Others (Specify) 77
J.2.	Main floor maternial<br><br>[Observation]	Concrete .... 1<br>Brick/Cement........ 2<br>Tin /CI sheet .... 3<br>Wood........ 4<br>Smoothed mud........ 5<br>Tile........ 6<br>Jute Stick .... 7<br>Bamboo/ Grass/straw .... 8<br>Others (Specify)........ 77
J.3.	Main exterior wall material<br><br>[Observation]	Concrete .... 1<br>Brick/Cement........ 2<br>Tin /CI sheet .... 3<br>Wood........ 4<br>Smoothed mud........ 5<br>Tile........ 6<br>Jute Stick .... 7<br>Bamboo/ Grass/straw .... 8<br>Others (Specify)........ 77
J.4.	Main roof material<br><br>[Observation]	Concrete .... 1<br>Brick/Cement........ 2<br>Tin /CI sheet .... 3<br>Wood........ 4<br>Smoothed mud........ 5<br>Tile........ 6<br>Jute Stick .... 7<br>Bamboo/ Grass/straw .... 8<br>Others (Specify)........ 77
J.5.	Do you have a garden where you grow vegetables and/or fruits?	Yes .... 1<br>No 0
J.6.	Does your household have electricity connection from national grid?	Yes .... 1<br>No 0
J.7.	Do you have any other kind of electric power? If yes, which type?	Generator .... 1<br>Solar........ 2<br>No other type 0
J.8.	What type of fuel does your household mainly use for cooking?	Electricity .... 1<br>LPG........ 2<br>Natural gas .... 3<br>Biogas .... 4<br>Kersone .... 5<br>Charcoal .... 6<br>Wood/ Straw/ Leaves .... 7<br>Animal dung .... 8<br>Others (Specify)____________ 77
J.9.	Do you have your own mobile phone?	Yes .... 1<br>No 0
J.10.	Do you have access to mobile phone?	Yes .... 1<br>No 0

J.11.	Are you a member of any community group/organization?	Yes .... 1<br>No .... 0>> module J
J.12.	Which community group/organization's member you are?	BRAC VO .... 1<br>BRAC Pllisomaj/union somaj................................. 2<br>Community clinic support group............................ 3<br>Community clinic management committee .... 4<br>Other (specify) 77

J.13. Household assets

I am now going to ask you about household items that are available in your household. For each item, please tell me if the item mentioned is available in your household? If yes, please tell me how many of each are available?

Asset	Asset code	How many are in usable Condition? (Number)	Asset	Asset code	How many are in usable Condition? (Number)
1	2	3	1	2	3
Metal cooking pots/pans	1		Microwave oven	18	
Bucket	2		Sewing machine	19	
Stove/Gas burner	3		Wall clock/wrist watch	20	
Plates/Pans	4		Camera	21	
Cup/mug	5		Bicycle	22	
Bed/Khat/Chowki	6		Motorcycle	23	
Mattress/blanket	7		Car/truck	24	
Table/ Chair	8		Rickshaw/Van	25	
Almirah	9		Bullock cart/Push cart	26	
Trunk / Suitcase	10		Boat	27	
Electric fan (Ceiling/Table)	11		Engine boat	28	
Table lamp	12		Phone/mobile phone	29	
Electric iron	13		Cow/buffalo	30	
Radio	14		Goat/sheep	31	
Audio cassette/CD player	15		Chicken/duck	32	
TV (color/black-white)	16		Other 1 (specify)…………….	33	
Refrigerator	17		Other 2 (specify)……………	34	

Water, sanitation and hygiene facilities

		Code	
J.14.	What is the main source of drinking water for members of your household?		Own tube well.. 1<br>Other's tube well.. 2<br>Community tube well .... 3<br>Ring Well/ Indara.. 4<br>Pond .... 5<br>River/canal... 6<br>Supply Water (piped) .... 7<br>Other (specify) 77
J.15.	What is the main source of water used by your household for cooking?		
J.16.	What is the main source of water used by your household for bathing?		
J.17.	What is the main source of water used by your household for washing utensils?		

J.18.	What kind of toilet facility do members of your household usually use?		Sanitary with flush (water sealed) .... 1<br>Sanitary without flush (water sealed) .... 2<br>Pucca/pit (without water sealed) .... 3<br>Kutcha/Hanging (fixed place) .... 4<br>open field .... 5<br>Other (specify) 77
J.19.	Do you share this toilet facility with other households?		Yes .... 1<br>No... 0

MODULE K. HOUSEHOLD FOOD SECURITY

Interviewer: For each of the following questions, consider what has happened *in the past 30 days.* For the questions "how often", the answer "Rarely" means 1-2 times, "Sometimes" means 3-10 times and "Often" more than 10 times

Sl. No	Questions	Code
K1	In the past 30 days did you worry that your household would not have enough food?	No.. 0>>K2<br>Yes 1
K1a	If "Yes", how often did this happen?	Rarely(1-2 times) ....1<br>Sometimes (3-10 times)...................2<br>Often (>10 times)............................3
K2	In the past 30 days were you or any household members not able to eat the kinds of foods you preferred because of a lack of resources?	No...0>>K3<br>Yes 1
K2a	If "Yes", how often did this happen?	Rarely(1-2 times) ....1<br>Sometimes (3-10 times)...................2<br>Often (>10 times)............................3
K3	In the past 30 days did you or any household member eat just a few kinds of food day after day because of a lack of resources?	No.. 0>>K4<br>Yes 1
K3a	If "Yes", how often did this happen?	Rarely(1-2 times) ....1<br>Sometimes ....2<br>Often (>10 times)............................3
K4	In the past 30 days did you or any household member eat food that you did not want to eat because of a lack of resources to obtain other types of food?	No...0>>K5<br>Yes 1
K4a	If "Yes", how often did this happen?	Rarely(1-2 times) ....1<br>Sometimes (3-10 times)...................2<br>Often..3
K5	In the past 30 days did you or any household member eat a smaller meal than you felt you needed because there was not enough food?	No...0>>K6<br>Yes 1
K5a	If "Yes", how often did this happen?	Rarely(1-2 times) ....1<br>Sometimes ....2<br>Often (>10 times)............................3
K6	In the past 30 days did you or any household member eat fewer meals in a day because there was not enough food?	No...0>>K7<br>Yes 1
K6a	If "Yes", how often did this happen?	Rarely(1-2 times) ....1<br>Sometimes (3-10 times)...................2<br>Often..3
K7	In the past 30 days was there ever no food at all in your household because there were no resources to get more?	No...0>>K8<br>Yes 1
K7a	If "Yes", how often did this happen?	Rarely(1-2 times) ....1<br>Sometimes (3-10 times)...................2<br>Often (>10 times)............................3
K8	In the past 30 days did you or any household member go to sleep at night hungry because there was not enough food?	No.. 0>>K9<br>Yes 1
K8a	If "Yes", how often did this happen?	Rarely(1-2 times) ....1<br>Sometimes ....2<br>Often..3
K9	In the past 30 days did you or any household member go a whole day without eating anything because there was not enough food?	No.. 0>>K10<br>Yes 1
K9a	If "Yes", how often did this happen?	Rarely(1-2 times) ....1<br>Sometimes (3-10 times)...................2<br>Often (>10 times)............................3

Food assistant/ support

K10	In the last one year, did anyone in the household receive food, cash or other tyoe of social assistance from anyone?	No.. 0>>K12<br>Yes 1
K11	If yes, what kind of assistance did you receive<br><br>(multiple responses possible)	Cash...1<br>Rice ....2<br>Wheat ....3<br>Other food......................................4<br>Other in-kind..................................5<br>Others (specify)..............................77
K12	Is anyone from the family currently receiving any micro credit loan?	Yes ....1<br>No........................ 0>> End this module
K13	If yes, where was the loan taken from?<br><br>(multiple responses possible)	BRAC..1<br>ASA...2<br>Caritas..3<br>Shonirbhar Bangladesh....................4<br>RDRS Bangladesh...........................5<br>Voluntary Organization for Social Development (VOSD).....................6<br>Bachte Shekha................................7<br>PKSF ....8<br>BRDB...9<br>Other NGO (specify) 77

MODULE L. WOMEN'S DIGNITY AND DECISION MAKING POWER

Sl. No	Questions		Code
L1	Now, I would like to have your opinion on some ideas regarding how people live within a household.<br>Please tell me if you agree or not with each of the following declarations.<br>There are people who say:		
1.	In a household, the man should take the important decisions.		No don't agree ....0<br>Yes, agree.....................................1<br>Don't know...................................99
2.	If the woman works outside home, her husband or partner should help her with the daily housework.		
3.	A husband should not let his wife work outside home, even if she would like to do it.		
4.	A woman has the right to express her opinion if she does not agree with what the husband or partner says.		
5.	A woman must accept that her husband or partner beats her in order to keep the family together.		
6.	It is better to send a son to school than a daughter.		
L2	Now, I would like to ask you some questions regarding your possessions. I am only asking these questions to better understand women's situation. (Don't forget, all that you tell us is confidential) Please tell me if you possess alone or together with somebody else one of the following things :		
1.	Land?		Yes alone ....1<br>Yes together ....2<br>Don't have 3
2.	This house or the house where you usually live?		
3.	Another house, apartment or room?		
4.	Animals like cows, horses, donkeys?		
5.	Small animals like hens, ducks, chickens, rabbits?		

<table>
<tr><th>Sl. No</th><th colspan="2">Questions</th><th>Code</th></tr>
<tr><td>6.</td><td>Gold jewelry?</td><td></td><td></td></tr>
<tr><td>L3</td><td colspan="3">WHICH FAMILY MEMBER DECIDES MOST OF THE TIME ABOUT THE FOLLOWING THINGS:</td></tr>
<tr><td>1.</td><td>Buying of food like rice, vegetables</td><td></td><td rowspan="15">Respondent 1
Husband of interviewee...................2
Interviewee and her Husband 3
Son or daughter..............................4
Son in law or daughter in law.........5
Brother or sister 6
Brother in law or sister in law.........7
Mother or father..............................8
Mother in law or father in law 9
Grandson or granddaughter 10
Other relatives 11
Not applicable.................................88
Do not know ....99</td></tr>
<tr><td>2.</td><td>Buying animal source foods (meat, fish, poultry, eggs)?</td><td></td></tr>
<tr><td>3.</td><td>Buying cooking oil</td><td></td></tr>
<tr><td>4.</td><td>Buying medicine for yourself</td><td></td></tr>
<tr><td>5.</td><td>Buying medicine for the children</td><td></td></tr>
<tr><td>6.</td><td>What food is prepared every day?</td><td></td></tr>
<tr><td>7.</td><td>If you have to work to earn money?</td><td></td></tr>
<tr><td>8.</td><td>Visiting other family members, friends or relatives?</td><td></td></tr>
<tr><td>9.</td><td>Seeing a doctor or visiting a dispensary when you are pregnant?</td><td></td></tr>
<tr><td>10.</td><td>Use of family planning methods?</td><td></td></tr>
<tr><td>11.</td><td>To eat nutritious food during pregnancy</td><td></td></tr>
<tr><td>12.</td><td>To take supplemental tablets during pregnancy</td><td></td></tr>
<tr><td>13.</td><td>To take rest every day for a certain time during pregnancy</td><td></td></tr>
<tr><td>14.</td><td>Whether or not you breastfeed the child and when to wean the child?</td><td></td></tr>
<tr><td>15.</td><td>What and how to feed the infant in his first year of life?</td><td></td></tr>
</table>

MODULE M. PHYSICAL AND MENTAL HEALTH OF THE RESPONDENT

No	Questions	Code
M1	Now, I would like to ask you about your health status.<br>If you compare your health status with other women in the area who have approximately the same age, how do you feel about your health?<br>(MAKE SURE THAT THIS QUESTION REFERS TO THE MOTHERS' HEALTH STATUS IN GENERAL, NOT RECENTLY)	1 | 2 | 3 | 4 | 5 | 6 | 7 | 8 | 9 | 10<br>Health is not good Health is good Health is very good
M2	**MATERNAL STRESS (SRQ 20)**<br>(I will ask if you faced a few problems within last 1 month)	

1	Do you often have headaches?		
2	Is your appetite poor?		
3	Do you sleep badly?		
4	Are you easily get frightened?		
5	Do your hands shake/tremble?		
6	Do you feel nervous, tense or worried?		
7	Is your digestion poor?		
8	Do you have trouble thinking clearly?		
9	Do you feel unhappy about life?		
10	Do you cry more than usual?		Yes ....1<br>No....................................0
11	Do you find it difficult to enjoy your daily activities?		
12	Do you find it difficult to make decisions?		
13	Is your daily work suffering?		
14	Are you unable to play a useful part in life?		
15	Have you lost interest in things?		
16	Do you feel that you are a worthless person?		
17	Has the thought of ending your life been on your mind?		
18	Do you feel tired all the time?		
19	Do you have uncomfortable feelings in your stomach?		
20	Are you easily tired?		

MODULE N. POSTNATAL FUNCTIONAL DISABILITY AND POSTPARTUM SIGNS/ SYMPTOMS

N1. Postnatal Functional Disability

Question	Within 1-7 days of giving birth			Within 8-14 days of giving birth			Within 15-30 days of giving birth			Within 31-42 days of giving birth		
	Yes.. 1<br>No....0	**<u>If yes,</u>**<br>Could do without difficulty….1<br>Could do with difficult…..2<br>**<u>If no,</u>**<br>Could not do at all.......3<br>Not permitted/ required to do......88		Yes.. 1<br>No....0	**<u>If yes,</u>**<br>Could do without difficulty….1<br>Could do with difficult…..2<br>**<u>If no,</u>**<br>Could not do at all.......3<br>Not permitted/ required to do......88		Yes.. 1<br>No....0	**<u>If yes,</u>**<br>Could do without difficulty….1<br>Could do with difficult…..2<br>**<u>If no,</u>**<br>Could not do at all.......3<br>Not permitted/ required to do......88		Yes.. 1<br>No....0	**<u>If yes,</u>**<br>Could do without difficulty….1<br>Could do with difficult…..2<br>**<u>If no,</u>**<br>Could not do at all.......3<br>Not permitted/ required to do......88	
Were you able to take care of the newborn baby?												
Were you able to feed the baby?												
Were you able to bathe the baby?												
Were you able to wash the baby's clothes?												
Were you able to prepare meals?												
Were you able to clean the house?												
Were you able to get water?												
Were you able to get to nearest health facility?												
Were you able to care for herself?												
Were you able to wash or bathe herself?												
Were you able to get dressed?												
Were you able to wash clothes?												
Were you able to use the toilet?												

N.2 Postpartum Signs/Symptoms

a. Think about duration after your delivery to 6 weeks (42 days) after birth. What signs/symptoms/diseases have you experienced? List the names of all signs and symptoms. Probe: Anything else? Until respondent says nothing else.

As the respondent recalls signs and symptoms, underline the corresponding signs and symptoms and write '1' in the column next to the signs and symptoms. If the signs and symptoms is not listed, write them in the box labeled 'others'. Once the respondent finishes recalling signs and symptoms, read each signs and symptoms where '1' was not written, ask the following question and write '1' if respondent says yes, '2' if no and '99' if don't know:

b. From delivery to 42 days after birth, did you experience the following symptoms?

c. For each of the reply with 1, continue to ask questions in the next two columns

		What symptoms did you experience after delivery?<br>Yes…………….1<br>No……………. 0<br>Don't know …..99	How long after delivery (in days)	For how many days (in days)
1.	Convulsions			
2.	Visual disturbance			
3.	Blindness			
4.	Coma or unconsciousness			
5.	Excessive vaginal bleeding			
6.	Abnormal bleeding from mucosa (mouth and/or ears)			
7.	Fever			
8.	Abdominal/uterine pain/tenderness			
9.	Foul smelling vaginal discharge/lochia			
10.	Productive cough and shortness of breath			
11.	Dysuria or flank pain			
12.	Headache			
13.	Neck stiffness			
14.	Continuous loss of urine and/or feces after delivery			
15.	Admission to hospital emergency unit			
16.	Laparotomy (includes hysterectomy, excludes caesarean section)			
17.	Use of blood/ blood products			
18.	Fatigue/weakness/lethargy			
19.	Swelling			
20.	Seizures			
21.	High blood pressure			
22.	Nausea/ Vomiting			
23.	Dizziness			
24.	Breast pain/engorgement/cracked or sore nipples/flat or inverted nipples			
25.	Others			

NEONATAL SIGNS/SYMPTOMS

a. Think about duration after your delivery to 4 weeks (28 days) after birth. What signs/symptoms/diseases has your child experienced? List the names of all signs and symptoms. Probe: Anything else? Until respondent says nothing else.

As the respondent recalls signs and symptoms, underline the corresponding signs and symptoms and write '1' in the column next to the signs and symptoms. If the signs and symptoms is not listed, write them in the box labeled 'others'. Once the respondent finishes recalling signs and symptoms, read each signs and symptoms where '1' was not written, ask the following question and write '1' if respondent says yes, '2' if no and '99' if don't know:

b. From delivery to 28 days after birth, did your infant experience the following symptoms?

c. For each of the reply with 1, continue to ask questions in the next two columns

	**Signs/ symptoms**	**What symptoms did your child experience after delivery?**<br>Yes…………….1<br>No……………. 0<br>Don't know …..99	**How long after delivery (in days)**	**For how many days (in days)**
	**N3_1**	**N3_2**	**N3_3**	**N3_4**
1.	Feeding difficulty			
2.	Breathing difficulty			
3.	Fast breathing			
4.	Severe chest-in drawing			
5.	Fits or convulsion			
6.	Movement only when stimulated or no movement at all			
7.	Fever			
8.	Hypothermia/low temperature			
9.	Umbilical cord infection			
10.	Skin infection			
11.	Jaundice/yellow color of skin			
12.	Vomiting/diarrhea			
13.	Others			

MODULE O. DOMESTIC VIOLENCE

	When two people marry or live together, they usually share both good and bad moments. I would now like to ask you some questions about your current and past relationships and how your husband / partner treats (treated) you. If anyone interrupts us I will change the topic of conversation. I would again like to assure you that your answers will be kept secret, and that you do not have to answer any questions that you do not want to. May I continue?			
O1	In relation to your (current or most recent) husband/partner, would you say it is generally true that he:	**Yes**	**No**	**Don't know**
	1. Tries to keep you from seeing your friends,	1	0	99
	2. Tries to restrict you from contacting your family of birth,	1	0	99
	3. Insists on knowing where you are at all times,	1	0	99
	4. Ignores you and treats you indifferently,	1	0	99
	5. Gets angry if you speak to another man,	1	0	99
	6. Is often suspicious that you are unfaithful,	1	0	99
	7. Expects you to ask his permission before seeking health care for yourself.	1	0	99

O2	Has your current husband/partner, or any other partner done the followings ever or in the last 12 months:	A. Has your husband/ partner ever…?	B. Has your husband/ partner in the last 12m…?
		Yes … 1<br>No … 0 >> **next Q**	Yes …………… 1<br>No ……………. 0
	1. Insulted you or made you feel bad about yourself?		
	2. Belittled or humiliated you in front of other people?		
	3. Done things to scare or intimidate you on purpose (e.g. by the way he looked at you, by yelling and smashing things)?		
	4. Threatened to hurt you or someone you care about?		
	5. Push you, shake you, or throw something at you?		
	6. Slap you?		
	7. Twist your arm or pull your hair?		
	8. Punch you with his fist?		
	9. Kick you, drag you or beat you up?		
	10. Try to choke you or burn you on purpose?		
	11. Threaten or attack you with a knife, gun, or any other weapon?		
	12. Physically force you to have sexual intercourse when you did not want to?		
	13. Have sexual intercourse you did not want to because you were afraid of what your partner or any other partner might do?		
	14. Forced you to do something sexual that you found degrading or humiliating?		

O3	During your last pregnancy, was there a time when you were beaten or physically assaulted by husband?	Yes.. 1<br>No.. 0

MODULE P. ANTHROPOMETRY

VERIFY HOUSEHOLD COMPOSITION TABLE: NOTE LINE NUMBER, NAME AND AGE OF RESPONDENT MOTHER AND HER RECENTLY DELIVERED BABY
NOTE: MAKES SURE ALL CHILDREN ARE MEASURED LYING DOWN

WEIGHT, HEIGHT OF RESPONDENT MOTHER							
Member ID (B01)	Name	DATE OF BIRTH<br>(DD MM YYYY)	AGE (IN YEARS)	WEIGHT (KG)	HEIGHT (CM)	CURRENT PREG-NANCY STATUS<br>Yes……..1<br>No………2<br>DK………99	RESULT<br>Measured...... 1<br>Absent.......... 2<br>Refused .... 3<br>Other………77
		[__][__]/ [__][__]/ [__][__][__][__]	[__][__]	[__][__].[__]	[__][__].[__]		
				[__][__].[__]	[__][__].[__]		

WEIGHT, HEIGHT OF THE CHILD

Member ID	Name	DATE OF BIRTH<br>(DD MM YYYY)	AGE (In months)	WEIGHT (KG)	HEIGHT (CM)	RESULT<br>Measured...... 1<br>Absent.......... 2<br>Refused .... 3<br>Other………77
		[__][__]/ [__][__]/ [__][__][__][__]	[__][__]	[__][__].[__]	[__][__].[__]	
				[__][__].[__]	[__][__].[__]	
